# Molecular-captured hot-electron detection in single-atom alloy antennas

**DOI:** 10.1093/nsr/nwaf174

**Published:** 2025-04-28

**Authors:** Yang Li, Yuanming Zhang, Zhaojian Zeng, Yong Chen, Zhonghua Li, Xiaoming Xu, Zhigang Zou, Zhipei Sun, Zhaosheng Li

**Affiliations:** Collaborative Innovation Center of Advanced Microstructures, National Laboratory of Solid State Microstructures, College of Engineering and Applied Sciences, Nanjing University, Nanjing 210093, China; Jiangsu Key Laboratory of Nano Technology, Nanjing University, Nanjing 210093, China; Collaborative Innovation Center of Advanced Microstructures, National Laboratory of Solid State Microstructures, College of Engineering and Applied Sciences, Nanjing University, Nanjing 210093, China; Jiangsu Key Laboratory of Nano Technology, Nanjing University, Nanjing 210093, China; Collaborative Innovation Center of Advanced Microstructures, National Laboratory of Solid State Microstructures, College of Engineering and Applied Sciences, Nanjing University, Nanjing 210093, China; Jiangsu Key Laboratory of Nano Technology, Nanjing University, Nanjing 210093, China; Collaborative Innovation Center of Advanced Microstructures, National Laboratory of Solid State Microstructures, College of Engineering and Applied Sciences, Nanjing University, Nanjing 210093, China; Collaborative Innovation Center of Advanced Microstructures, National Laboratory of Solid State Microstructures, College of Engineering and Applied Sciences, Nanjing University, Nanjing 210093, China; Jiangsu Key Laboratory of Nano Technology, Nanjing University, Nanjing 210093, China; Collaborative Innovation Center of Advanced Microstructures, National Laboratory of Solid State Microstructures, College of Engineering and Applied Sciences, Nanjing University, Nanjing 210093, China; Jiangsu Key Laboratory of Nano Technology, Nanjing University, Nanjing 210093, China; Collaborative Innovation Center of Advanced Microstructures, National Laboratory of Solid State Microstructures, College of Engineering and Applied Sciences, Nanjing University, Nanjing 210093, China; Jiangsu Key Laboratory of Nano Technology, Nanjing University, Nanjing 210093, China; QtF Centre of Excellence, Department of Electronics and Nanoengineering, Aalto University, Aalto FI-00076, Finland; Collaborative Innovation Center of Advanced Microstructures, National Laboratory of Solid State Microstructures, College of Engineering and Applied Sciences, Nanjing University, Nanjing 210093, China; Jiangsu Key Laboratory of Nano Technology, Nanjing University, Nanjing 210093, China

**Keywords:** hot-electron detection, *in situ* photoexcitation instrument, single-atom alloy antennas, atomic-scale photoelectric effect

## Abstract

Hot electrons are ubiquitous in diverse physical and chemical processes, since they are involved in the energy transfer of elementary processes such as adsorption, diffusion and desorption of reactants. However, hot electrons have short lifetimes (∼few fs) and small mean free paths (∼<10 nm), which are inherently difficult to detect via conventional *in situ* pumping techniques. Here, we designed a spectrally tunable photoexcitation desorption analyser, a tool for tracking hot-electron generation, which enables mechanistic studies of hot-electron generation and transfer in single-atom alloy antennas in real time under flow conditions by a variety of molecular probes (CO, CO_2_ and various hydrocarbons). Long-lived hot electrons arise because electrons with discrete energy levels spaced by several hundred meV in individual atoms cannot relax to form phonons. Furthermore, we utilize the hot electrons generated by single-atom alloy antenna-modified photocatalysts under illumination to produce green syngas from carbon dioxide and water, achieving an efficiency one order of magnitude higher than traditional powder photocatalysis. Our discovery provides an unprecedented perspective for the detection of hot-electron generation and has implications for future advancements in nanophotonics.

## Teaser

A tunable photoexcited desorption analyser reveals real-time hot-electron dynamics in single-atom alloy antennas, offering new insights into nanoscale energy transfer.

## INTRODUCTION

The advancement of ultrafast spectroscopy and low-dimensional synthesis technologies has enhanced our understanding of the generation and transfer of hot electrons via the macroscopic photoelectric effect. For example, when photons act on a metal surface, the thermalized electrons can interact with the phonons on the metal surface because of their relatively wide energy range distribution and low movement speed. The time scale of this electron–phonon scattering is within the picosecond (ps) scale. Once hot electrons are generated, they undergo electron–electron and electron–phonon interactions within their mean free path, ultimately transforming into low-energy electrons [1]. This process occurs in an extremely short time, making detection very difficult, and the resulting low-energy electrons exhibit reduced reaction sensitivity, which limits their practical application. Therefore, there is an urgent need to detect the generation and transfer of hot electrons in close proximity to the actual reaction process for a future net-zero chemical industry via photon utilization.

Moreover, photoelectric effects caused by the interaction of light and matter [1–3] have been thoroughly investigated because of their crucial function in harnessing photon energy [1–7]. The photoelectric effect of metals or alloys is utilized to facilitate various important light-driven reactions, including methanol preparation reactions [8], syngas production reactions [9], reverse water gas reactions [10], Sabatier reactions [11] and Holy Grail reactions [12]. Significant differences in reaction activity have been observed due to size effects, but there is no unified understanding of the size–performance mechanism underlying these differences, owing primarily to the difficulty in tracking molecular states under light excitation. Because of the lack of specialized tools for thorough examination, it is unclear whether the hot electrons generated by the atomic-scale photoelectric effect induce mechanisms that differ from those of traditional theories. Elucidating the generation and transmission mechanism of hot electrons via the atomic-scale photoelectric effect can provide critical guidance for vastly improving these significant light-driven reactions. If a quantitative relationship between hot electrons at the atomic scale and molecular interactions can be established from the perspective of analytical chemistry, it will provide a new dimension for the precise control of reactivity.

Here, we created an *in situ* photoexcitation instrument to detect hot-electron generation and transfer in addition to quantitatively identifying reactive sites. We determined that the photoelectric effect still exists by quantifying the contribution of hot electrons in a single-atom alloy antenna, which is a highly active and selective alloy catalyst that has attracted intensive interest [13]. Given that FeVO_4_ possesses a bandgap of ∼2.1 eV and demonstrates potential for photocatalytic CO_2_ reduction, we selected the FeVO_4_ photocatalyst (denoted as FeVO) and single-atom FeV alloy antenna-modified FeVO_4_ photocatalyst (denoted as FeV@FeVO) as subjects for investigation to facilitate a direct comparison of the contributions of photoelectrons and hot electrons to molecular activation. Furthermore, light-driven syngas production over FeV@FeVO was chosen as a model reaction, motivated by the goal of efficient photon utilization, since syngas is an ideal ‘future fuel’ applicable for both direct use in internal combustion engines and for the Fischer–Tropsch synthesis of liquid fuels [14,15]. The syngas yield was one order of magnitude greater than that of the powder photocatalysis system without any sacrificial agents, due to the atomic-scale photoelectric effect of the single-atom alloy antennas in the FeV@FeVO, which efficiently activated CO_2_ and H_2_O molecules through long-lived hot electrons.

## RESULTS AND DISCUSSION

### Atomic-scale photoelectric effect

**Figure 1.**
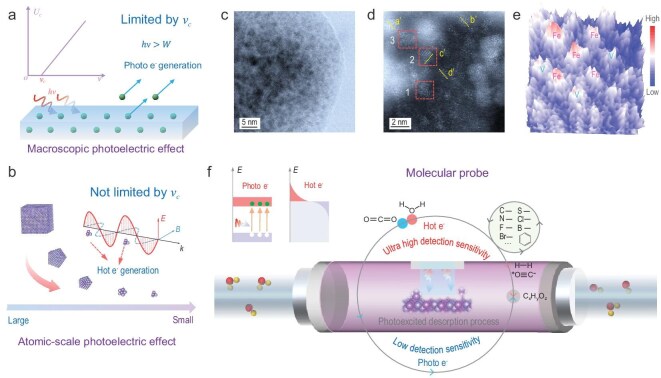
Proposal and utilization of the atomic-scale photoelectric effect. (a) Macroscopic photoelectric effect of bulk metals. (b) Atomic-scale photoelectric effect. (c, d) High angle angular dark field-scanning transmission electron microscopy (HAADF-STEM) images of FeV@FeVO at high magnification. (e) 3D intensity surface plot shown in dashed region 1 of the image in (d). (f) Schematic diagram of the *in situ* photoexcitation desorption (PED) process to reveal the scalability of photon utilization of the atomic-scale photelectric effect.

The classical mechanism for the photoelectric effect emphasizes that photoelectrons can be excited only when the incident photon energy exceeds the work functions of the materials (Fig. 1a). However, the initial theory of the photoelectric effect overlooked the impact of material size. At the atomic scale, the photoelectric effect is not restricted by the material cut-off frequency since the electromagnetic field produced by incident light significantly enhances the internal electron excitation process (Fig. 1b). Therefore, we propose the atomic-scale photoelectric effect. When the size of an object is reduced to the atomic scale, ground-state electrons not only absorb the energy from incident photons but also have their excitation processes enhanced by the electromagnetic field of the incident light. This enhancement leads to excited electrons possessing higher energy (hot electrons), and hot electrons exhibit greater sensitivity in both detection and activation processes.

To demonstrate the atomic-scale photoelectric effect, we first synthesized an optimal single-atom alloy antenna-modified photocatalyst (FeV@FeVO) alongside a reference FeVO_4_ photocatalyst for our research model. Several island-shaped alloys are exposed on the surfaces of the FeV@FeVO samples (Fig. 1c and d, Figs S1 and S2). Three regions in Fig. 1d were selected for analysing the elemental composition of the single-atom alloys. Alloys in regions 1, 2 and 3 consist primarily of equal molar ratios of Fe and V (Fig. 1e, Figs S3 and S4). The linear distribution diagram shown in the other parts of Fig. 1d further confirms that the single-atom alloys are primarily composed of Fe and V in roughly the same proportions (Fig. S5) [16]. Additionally, the Fe phase appears in the X-ray diffraction (XRD) patterns (Fig. S6). K-edge X-ray absorption near edge structure (XANES) spectroscopy revealed that the typical valence states of Fe in the FeVO and FeV@FeVO samples were ∼2.5 and 0.8, respectively. The single-atom FeV@FeVO samples have weak peaks at ∼2.25 Å and 2.64 Å attributed to the Fe–Fe and Fe–V paths, respectively, whereas these two peaks are absent in the FeVO samples (Figs S7–S9). According to Figs S10 and S11, the typical valence state of V in the FeVO sample is ∼4.5, whereas it is ∼0.9 in the single-atom FeV@FeVO sample [17]. Furthermore, the Fourier-transform extended X-ray absorption fine structure (FT-EXAFS) spectra (Fig. S12) revealed faint peaks at ∼2.64 Å that correspond to the Fe–V paths, across the FeV@FeVO sample. Wavelet transform (WT)-EXAFS analysis involving Fe and V and X-ray photoelectron spectroscopy (XPS) of FeVO and FeV@FeVO (Figs S13–S19) also supported these results [17,18]. Therefore, it can be confirmed that a single-atom alloy composed of Fe and V exists in the FeV@FeVO sample.

To detect the atomic-scale photoelectric effect and promote practical applications, a photoexcitation desorption analyser was developed (Fig. 1f and Fig. S20). The single-atom alloys function as efficient antennas for hot electron generation. The core cavity of the photoexcitation instrument enables reversible switching between light and dark states. By employing diverse molecular probes to capture signals, we achieve direct analysis of the photoexcited hot electron generation process (Fig. 1f). To be precise, a material's unique coordination environment results in relatively strong interactions with gas molecules at the atomic scale. Under light excitation, many hot electrons are generated in the vicinity of atomic-scale materials, and these hot electrons play a pivotal role in greatly accelerating the activation and desorption of gas molecules. By detecting photoexcited desorption signals, we can obtain more comprehensive insight into the generation and activation dynamics of hot electrons. The atomic-scale photoelectric effect may broaden green synthesis pathways, such as highly anticipated syngas production reactions and free radical reactions.

**Figure 2.**
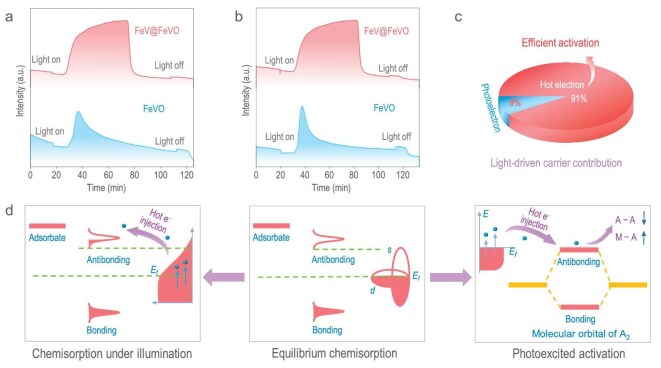
Hot-electron generation via the atomic-scale photoelectric effect. (a, b) Photoexcited desorption test of CO_2_ (a) and CO (b) as probe molecules over the FeVO and FeV@FeVO samples. (c) Quantification of light-driven carrier contributions. (d) Schematic illustrations of the photoexcited hot electron mechanism from a molecular orbital perspective in the atomic-scale photoelectric effect.

Under illumination, the features of CO_2_ desorption on the FeV@FeVO surface and reference FeVO sample were examined (Fig. 2a). For the FeV@FeVO sample, a continuous broad peak was observed, indicating superior performance due to the relatively stable signal. This suggests that residual CO_2_ is continuously present through chemical adsorption. Notably, there are 2.05 mmol g^−1^ photoactivation sites for CO_2_ on the surface of FeV@FeVO, which is ∼10 times greater than that for the FeVO sample (0.203 mmol g^−1^). Whereas the photoactive sites on the FeV@FeVO surface result from the combined contributions of photoelectrons and hot electrons, the photoactive sites on the FeVO surface are mainly from photoelectrons alone.

To verify the atomic-scale photoelectric effect, we use CO as another molecular probe. During the experiment, the number of CO desorption sites was estimated via the photoexcited desorption property of CO, which also helped to clarify how the single-atom alloy interacted with the support when illuminated. The number of photodesorption sites for CO on the FeV@FeVO surface is 2.564 mmol g^−1^, which is ∼11 times greater than the number of photodesorption sites (0.234 mmol g^−1^) for CO on the FeVO surface (Fig. 2b). This result also demonstrated how hot electrons from single-atom alloys contribute to CO desorption. Furthermore, because CO is a polar molecule, the electron clouds surrounding single-atom alloys strongly polarize it, which causes a sizable amount of CO to stick to their surface. However, strong interactions between the support and single-atom alloys prevent CO from adhering strongly to the surface of the single-atom alloy antenna and poisoning the catalyst. CO will absorb the energy required to detach from the bonds of the single-atom alloy and desorb as a reaction product when exposed to light. On the basis of these results, we can conclude that our *in situ* photoexcitation experiments on single-atom alloy antennas with molecular probes reveal that hot electrons contribute ∼10 times greater activation than photoelectrons (Fig. 2c).

Utilizing molecular orbital theory [19], we elucidate the mechanism of hot-electron-induced molecular activation. As illustrated in Fig. 2d, upon achieving adsorption equilibrium on the catalyst surface, bonding orbitals (occupied) and antibonding orbitals (unoccupied) are established. Following photoexcitation, the single-atom alloy catalyst produces a substantial number of hot electrons, which effectively populate the antibonding orbitals of the adsorbed molecules, resulting in their swift desorption. Consequently, the number of desorbed molecules serves as an indicator for quantifying hot electrons. Additionally, hot electrons facilitate chemical reactions by promoting dissociative adsorption. Specifically, they weaken the intramolecular bond (A–A) of the adsorbed species (A_2_) while enhancing the interaction between the catalyst and the adsorbate (M–A). This dual effect markedly improves molecular dissociation and activation.

**Figure 3.**
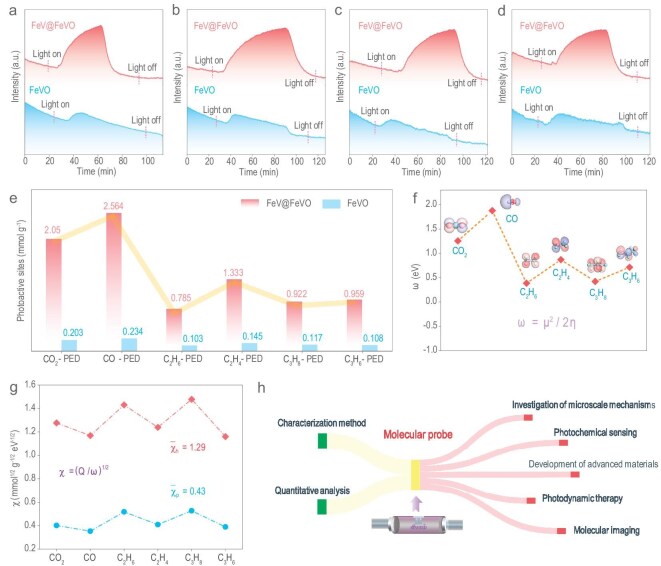
Quantitative criteria for hot electrons via molecular probes. (a–d) Photoexcited desorption test of C_2_H_6_ (a), C_2_H_4_ (b), C_3_H_8_ (c) and C_3_H_6_ (d) as probe molecules over the FeVO and FeV@FeVO samples. (e) Statistical comparison of photoactive sites between FeV@FeVO and FeVO. (f) Electrophilicity of all the molecules, *ω* = *μ*^2^/2*η, μ* = (*E*_LUMO_ + *E*_HOMO_)/2, *η* = *E*_LUMO_ − *E*_HOMO_. (g) Sensitivity of hot electrons and photoelectrons measured by a molecular probe, where χ represents the sensitivity of the molecular probe, where Q represents the photoactive site statistics, and where ω represents the electrophilicity of the molecules. (h) Potential applications of molecular probes based on photoexcitation experiments.

Additional tests on hydrocarbon compounds (C_2_H_6_, C_2_H_4_, C_3_H_8_ and C_3_H_6_) under identical conditions were conducted to better comprehend the dynamics of hot electrons in the desorption and activation of gas molecules. The number of photoactivation sites on the FeV@FeVO sample is significantly greater than that on the FeVO sample, as shown in Fig. 3a–e. This finding supports the important role of hot electrons in the photoactivation process. CO_2_ and CO exhibit higher degrees of unsaturation and electrophilicity than hydrocarbon compounds do, resulting in more pronounced interactions with hot electrons. Moreover, owing to the nucleophilic nature of C_2_H_6_ and C_3_H_8_, the involvement of hot electrons in their activation process is diminished. C_2_H_4_ and C_3_H_6_ possess electrophilic C=C orbitals that are more conducive to interacting with hot electrons than are C_2_H_6_ and C_3_H_8_.

The term *μ*^2^/2*η*, denoted as *ω*, can be viewed as a gauge of the electrophilicity of the molecules [20]. CO exhibited the highest electrophilicity, followed by CO_2_. The electrophilicity of C_2_H_4_ and C_3_H_6_ hydrocarbon compounds is greater than that of C_2_H_6_ and C_3_H_8_ (Fig. 3f, Fig. S21 and Table S1). A greater electrophilicity enhances the affinity of a molecule for a single-atom alloy catalyst, increasing the likelihood of adsorption on the catalyst's surface. When exposed to light, the system will exhibit increased desorption and activation capabilities, which is consistent with the findings of the photoexcited desorption test. According to the above results, we further confirmed that the number of photoactive sites on the surface of FeV@FeVO is significantly greater than that on the surface of FeVO. The hot electrons produced by the photoelectric effect in a single-atom alloy have more focused energy, leading to a greater contribution to the activation of inert molecules. We can assess the electrophilicity and nucleophilicity of compounds by quantitatively analysing the impact of hot electrons on the activation process.

To further evaluate the effectiveness of the molecular probes, we defined the molecular detection sensitivity (*χ* = (*Q*/*ω*)^1/2^). As shown in Fig. 3g, by examining the detection of single-atom alloy catalysts with various molecules, we found that the detection sensitivity of hot electrons is 1.29 mmol^1/2^ g^−1/2^ eV^−1/2^, which is three times greater than that of photoelectrons (0.43 mmol^1/2^ g^−1/2^ eV^−1/2^). This further demonstrates the high activation capability of hot electrons for probe molecules. Through the development of molecular probes and quantitative methodologies, we can enable versatile applications across multiple domains for molecular probes, including the investigation of microscale mechanisms, photochemical sensing, photodynamic therapy, the development of advanced materials and molecular imaging (Fig. 3h).

### Deciphering the atomic-scale photoelectric effect

**Figure 4.**
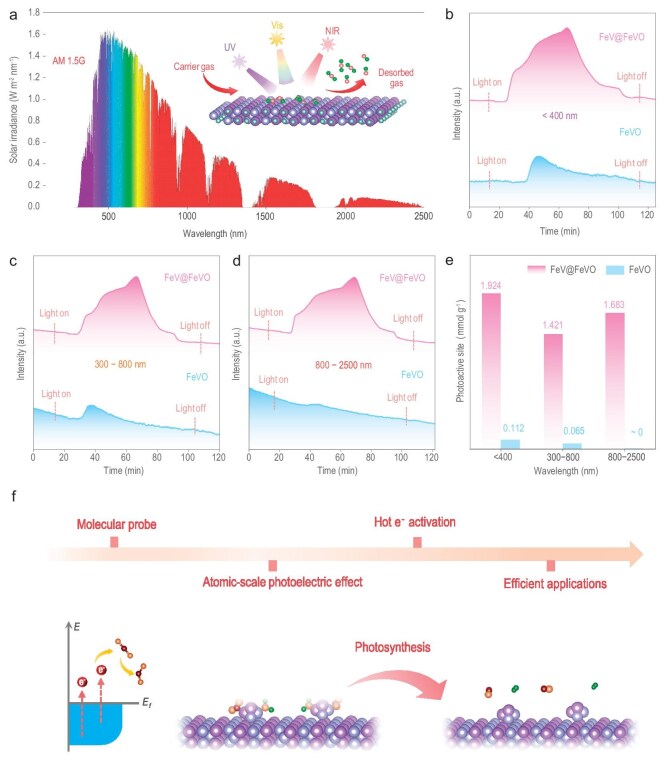
Photoexcited probe reactions under various wavelength regions. (a) Solar spectrum. Illustration represents a schematic diagram of the photoexcited desorption process under various wavelength regions. (b–d) Photoexcited CO_2_ desorption test over the FeV@FeVO and FeVO samples under irradiation at the following wavelength regions: <400 nm (b), 300–800 nm (c) and 800–2500 nm (d). (e) Photoactive site statistics for the FeV@FeVO and FeVO samples under various wavelength regions. (f) Schematic diagram of extending the utilization of hot electrons to a photosynthesis system.

To further confirm the role of hot electrons generated by the photoelectric effect of a single-atom alloy in the activation process of CO_2_, CO_2_ desorption under illumination by various wavelength regions (with the same intensity) was performed (Fig. 4a). Excited by ultraviolet light, the photoactivation sites for CO_2_ on the surfaces of FeV@FeVO and FeVO are 1.924 and 0.112 mmol g^−1^, respectively. This finding indicates that both photoelectrons and hot electrons in the FeV@FeVO samples can participate in the activation of CO_2_ simultaneously, with hot electrons being the primary contributors (Fig. 4b). Moreover, the activation of CO_2_ by photoelectrons decreases as the wavelength of the excitation light increases (Fig. 4c and d). In particular, photoelectrons rarely participate in the activation of CO_2_ when the incident light is in the infrared region (Fig. 4d). Experiments at various wavelengths demonstrated that the atomic-scale photoelectric effect is not restricted by the cut-off frequency and that the electron excitation process is strongly influenced by the electromagnetic field of the incident light. This method challenges the existing comprehension of light–matter interactions and offers theoretical and experimental insights into the atomic-scale photoelectric effect. Furthermore, this method offers a strong way to determine the impact of photoelectrons on their varying reactivities to different wavelength regions (Fig. 4e). Through characterization of single-atom alloy antennas using molecular probes (CO, CO_2_ and various hydrocarbons), we identified fundamental differences between atomic-scale and macroscopic photoelectric effects. The atomic-scale photoelectric effect generates abundant hot electrons that demonstrate enhanced activation efficiency toward probe molecules. Our findings demonstrate significant potential for scaling these hot electrons generated through atomic-scale photoelectric effects to photosynthesis (Fig. 4f).

### Imitating natural photosynthesis for practical applications

**Figure 5.**
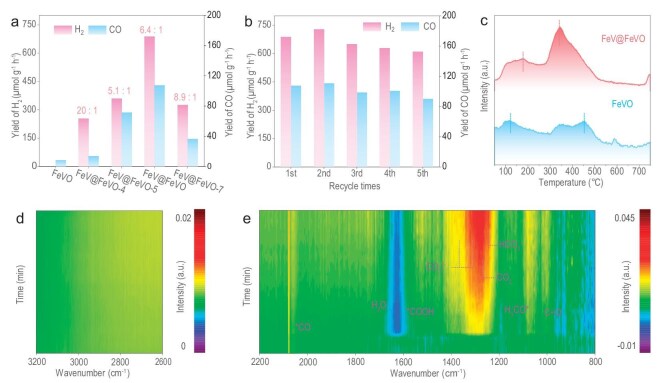
Photosynthesis for green syngas dominated by hot electrons. (a) Photosynthesis performances of the synthesized samples (FeVO, FeV@FeVO-4, FeV@FeVO-5, FeV@FeVO and FeV@FeVO-7). (b) Stability evaluating for syngas generation over FeV@FeVO. (c) Temperature-programmed desorption (TPD) of CO_2_ over FeV@FeVO and FeVO samples. (d, e) *In situ* diffuse reflectance infrared Fourier transform spectroscopy (DRIFTS) of FeV@FeVO via imitating natural photosynthesis (the test duration is 30 minutes).

FeV@FeVO and the reference FeVO photocatalyst were used to produce green syngas from carbon dioxide and water under illumination. The FeVO sample produced a small amount of CO (7.6 μmol g^−1^ h^−1^) (Fig. 5a), whereas the FeV@FeVO sample produced a significant amount of CO and H_2_ reduction products, as well as O_2_ and H_2_O_2_ oxidation products (Fig. 5a and Fig. S22). The proportion of syngas (CO + H_2_) can be regulated by modifying the synthesis conditions of the FeV@FeVO samples. The rates of CO and H_2_ over the FeV@FeVO sample reached ∼107 and 684.8 μmol g^−1^ h^−1^, respectively (Fig. 5a and Table S2). Furthermore, the FeV@FeVO sample exhibited long-term stability for CO_2_ reduction (Fig. 5b). A large amount of CO_2_ was strongly adsorbed on the FeV@FeVO surface (Fig. 5c), which suggested that there were sufficient CO_2_ adsorption sites on its surface, facilitating CO_2_ activation. Single-atom alloys have a looser confinement of the light field, resulting in a longer lifetime of excited hot electrons [21]. The use of hot electrons in single-atom alloys is anticipated to greatly increase photosynthesis efficiency. Additionally, a desorption peak at ∼300°C (Fig. S23) demonstrated that a significant amount of CO may be desorbed on the FeV@FeVO surface [22], indicating that the FeV@FeVO sample is conducive to the production of CO. The formation of alloy has a greater impact on the density of electronic states of FeV@FeVO than on that of the FeVO sample (Fig. S24), which also enhances the light absorption of the catalyst (Fig. S25). There are more oxygen vacancies on the surface of FeV@FeVO than on that of FeVO (Fig. 5d), encouraging the activation of CO_2_ and H_2_O. In particular, when hydrogen is used as the reducing agent instead of water in this reaction, almost no production occurs in the system (Fig. S26), demonstrating the importance of water throughout the entire reaction. The contact angle of FeV@FeVO was 6.7°, which is much smaller than that of FeVO (63.8°), indicating that the FeV@FeVO surface became hydrophilic (Figs S27–S30) [23]. Steady-state photoluminescence (PL) spectra and average fluorescence lifetime suggest a better charge separation capability with reduced recombination rates for the FeV@FeVO sample (Figs S31 and S32). These findings reveal the potential advantages of combining single-atom alloy antennas with semiconductor materials [24].

The absence of C−H stretching vibrations between 3000 and 3200 cm^−1^ (Fig. 5d) indicates the high selectivity of the CO product [25]. The peaks at 1023 cm^−1^ and 2090 cm^−1^ correspond to the stretching vibrations of the C−O bond and *CO, respectively (Fig. 5e) [26]. The intermediates CO_3_^2−^, HCO_3_^−^ and CO_2_^−^ are primarily indicated by signal peaks at 1246 cm^−1^, 1296 cm^−1^ and 1328 cm^−1^, respectively, reflecting the dynamic evolution of CO_2_ adsorption and activation [27]. The principal symbol in this reaction is represented by the signal at 1596 cm^−1^ of the *COOH intermediate [28,29]. The activation of CO_2_ produces CO_3_^2−^, HCO_3_^2−^ and CO_2_ intermediates, whereas the activation of H_2_O generates *H intermediates. These *H intermediates facilitate the reduction of CO_3_^2−^, HCO_3_^−^ and CO_2_^−^ intermediates, leading to the generation of *COOH and *CO intermediates. Finally, the surface of the FeV@FeVO desorbs H_2_ and CO. Additionally, we examined other FeV@FeVO samples synthesized under different conditions using *in situ* DRIFTS. While maintaining vibration peaks at identical positions to the optimal sample, these samples exhibited time-dependent variations in signal intensity. This indicates that synthesis condition modifications can tune the syngas production ratios, as corroborated by chromatographic analysis (Figs S33–S35). FeVO was subjected to an *in situ* DRIFTS under the same conditions, and the vibration stretching peaks of different intermediates were not clearly visible, possibly due to the absence of activation sites on the surfaces of the intermediates, thus resulting in poor photocatalytic activity for CO_2_ (Fig. S36).

The FeV@FeVO sample provides CO_2_ with a charge of 0.744 eV, which breaks the C−O bond and accelerates subsequent activation (Figs S37 and S38). In addition to the activation of CO_2_, a *COOH intermediate is created [30,31]. Charge is transferred from FeV@FeVO to *COOH in this phase, giving *COOH a charge of 0.406 eV. The *COOH intermediate subsequently changes to the *CO intermediate. During the interaction with FeV@FeVO, *CO transfers its charge to the material, leading to the conversion of *CO into CO.

During the adsorption and activation of CO_2_, which is the core step of the reaction [32,33], 2.663 eV is released over FeV@FeVO, whereas only 0.023 eV is released over FeVO (Fig. S39). This result indicates that FeV@FeVO has a greater ability to activate CO_2_ than does FeVO. Furthermore, *H produced by the activation of H_2_O is adsorbed primarily on the Fe site of FeV@FeVO, and each *H has a charge of 0.318 eV, allowing *H to participate in the CO_2_ reduction reaction (Fig. S40). Furthermore, the activation of H_2_O on FeV@FeVO releases 1.056 eV, which is significantly greater than that on FeVO (Fig. S41).

Integrating the experimental evidence with results from theoretical calculations, an overall understanding of the reaction path is obtained. The single-atom alloys on FeV@FeVO are the main active sites, and CO_2_ and H_2_O can be activated easily in their vicinity (Fig. S42). The *H generated by the activation of H_2_O acts as a reducing agent to accelerate the breaking process of the C−O bond of CO_2_, and *COOH and *CO intermediates subsequently form. After that, *CO can further be reduced by *H, and the syngas (CO and H_2_) will be released.

By utilizing atomic-scale photoelectric effects, the photon utilization efficiency can be regulated, and high-value production costs can be reduced. For example, multicarbon products such as olefins, alcohols, aldehydes, ketones and ethers prepared from syngas have significant applications in both the energy sector and daily life. Traditional heat-driven methods not only increase production costs but also have considerable environmental impacts [34]. However, introducing photon substantially reduces overall production costs and enhances production efficiency, making it an efficient approach for the green synthesis of syngas (Fig. S43).

## CONCLUSION

The reported self-designed photoexcited desorption analyser enables the identification and kinetic resolution of hot-electron generation. By combining experimentally determinable active sites with theoretical molecular electrophilicity, we can confirm that this consistency is due primarily to the energy transfer of hot electrons between molecules. We observe that the atomic-scale photoelectric effect in a single-atom alloy is distinct from the macroscopic photoelectric effect. The atomic-scale photoelectric effect can generate substantial and long-lived hot electrons, enabling reactivity during miscellaneous photosynthesis, independent of the material's work function. As a theoretical demonstration, a single-atom alloy antenna-modified photocatalyst showed a 10-fold improvement in the yield of tunable syngas from CO_2_ and H_2_O without any sacrificial agent. The rational design of these green synthesis routes using a photoexcited desorption analyser has the potential to be extended to other systems. These results hold promise for further boosting the efficiency of nanophotonics in green synthesis, ultimately overcoming reaction limitations.

## Supplementary Material

nwaf174_Supplemental_File
