# Supplementary material for: Molecular-captured hot-electron detection in single-atom alloy antennas
Source: Natl Sci Rev. 2025 Apr 28;12(6):nwaf174. doi: 10.1093/nsr/nwaf174 (PMC12125980; doi:10.1093/nsr/nwaf174)
Supplement: nwaf174_Supplemental_File [file nwaf174_supplemental_file.pdf]

Supplementary Materials for

Molecular-captured hot-electron detection in single-atom alloy antennas

Yang Li[1, 2, †], Yuanming Zhang[1, 2, †], Zhaojian Zeng[1, 2], Yong Chen[1], Zhonghua Li[1, 2], Xiaoming Xu[1, 2], Zhigang Zou[1, 2], Zhipei Sun[3], Zhaosheng Li[1, 2, *]

[1]Collaborative Innovation Center of Advanced Microstructures, National Laboratory of Solid State Microstructures, College of Engineering and Applied Sciences, Nanjing University, 22 Hankou Road, Nanjing 210093, China

[2]Jiangsu Key Laboratory of Nano Technology, Nanjing University, 22 Hankou Road, Nanjing 210093, China

[3] QTF Centre of Excellence, Department of Electronics and Nanoengineering, Aalto University, Espoo, Finland

*Corresponding Author. Email: zsli@nju.edu.cn

[†]These authors contributed equally to this work.

Materials and Methods

Preparation of the samples

In the experiment, 1.0 mmol of $Fe(NO)_3{\cdot}9H_2O$ was added to a 1.0 mmol of NH_4VO_4 aqueous solution, after which a yellow turbid solution formed. The yellow turbid solution was then evaporated to dryness at 95 °C to form the precursor. Subsequently, the precursor was baked at 350 °C for 2 h and thoroughly ground, and finally, the $FeVO_4$ (denoted as FeVO) samples were obtained after annealing at 650 °C for 2 h. Moreover, 1 g of each FeVO sample was mixed with 1 g of $NaBH_4$ and then sintered at 600 °C for 2 h. After washing and drying, a single-atom FeV alloy antennas modified $FeVO_4$ (denoted as FeV@FeVO) photocatalyst was obtained. By changing the sintering temperature to 400°C, 500°C and 700°C, we obtained FeV@FeVO-4, FeV@FeVO-5, and FeV@FeVO-7, respectively.

Characterizations

X-ray diffraction (XRD) patterns of all the samples were carried out with a Shimadzu LabX-XRD-6000 instrument with Cu Kα radiation (40 kV, 40 mA). High-angle annular dark-field scanning transmission electron microscopy (HAADF-STEM)

images were taken using a Titan Cubed Themis G2 300. The binding energy and valence band spectra of the films were measured via X-ray photoelectron spectroscopy (XPS; UIVAC-PHI, Japan). UV–visible (UV–vis) absorption spectra of all the samples were measured by a spectrophotometer (Shimadzu UV-2550, Japan). The surface morphology of all the samples was observed by scanning electron microscopy (SEM, ZEISS ULTRA 55, Germany) and high-resolution transmission electron microscopy (HRTEM, JEM 200CX TEM apparatus, Japan). Photoluminescence (PL) and time-resolved photoluminescence (TRPL) measurements were performed on the films using a Horiba Fluorolog3 spectrofluorometer. The excitation wavelength was 470 nm, and the probed wavelength was 530 nm. The X-ray absorption spectra (XAS), including XANES and EXAFS at the Fe K-edge and V K-edge, of the samples were measured at beamline 14 W of the Shanghai Synchrotron Radiation Facility in China.

Imitating natural photosynthesis experiments

The photocatalytic syngas production of all the samples was performed in a quartz-material reactor (CEL-APR100H-3, CEAuLight Co., Ltd., China). High-purity CO_2 served as the reactant, and a little quantity of water was fed as a reducing agent into the reaction system overnight in dark to allow full contact between the reactants and the catalyst. Then, a 300 W xenon lamp (CEL-PF300-T8, CEAuLight Co., Ltd., China) was used as a solar simulator to perform photocatalytic syngas production on the reaction system. A gas chromatograph (GC-8890, Agilent Technologies, Inc., USA) was used to analyse the properties of the products. We used a 2.5 ml syringe to transfer the products from the reactor to the gas chromatograph for analysis during the reaction. Fourier transform infrared spectroscopy (FT-IR, Nicolet iS50) was used to analyse the intermediates during the catalytic reaction. First, the catalysts were fully purged in an argon stream. Afterward, the samples were fully exposed to a mixed gas (H_2/CO_2 = 1:1) flow of 15 mL min^{-1} for 60 min in dark and then maintained under illumination by a Xe lamp (CEL-PF300-T8, CEAuLight Co., Ltd., China) for 1 h.

The temperature-programmed CO_2 desorption (CO_2-TPD) of all the samples was tested by a TPD apparatus. All the catalysts were first purged in a stream of argon at 150 °C for 2 h and then cooled to 25 °C. Subsequently, the saturated adsorption of CO_2 on the catalyst was completed at 50 °C for 50 min, after which the physisorbed molecules were removed by sweeping with argon. Finally, the catalysts were uniformly heated at a rate of 10 °C min^{-1} in an argon gas stream. CO-TPD was performed on the same apparatus used for CO_2-TPD.

Photoexcited desorption test

We designed a photoexcited desorption analyser. In detail, first, the sample was fully in contact with the measured gas (such as CO_2 or CO); then, the carrier gas was turned on, and the gas that had been weakly absorbed on the surface of the sample was removed. After a period of time, the light is turned on, and the properties of gas absorption can be collected by the detector.

Computational details

We used the Vienna ab initio simulation package to execute all the calculations. The generalized gradient approximation in the Perdew–Burke–Ernzerh of scheme was used for the exchange correlation functional. The proposed Hubbard U correction was considered to modify the strong on-site Coulomb repulsion between localized 3*d* electrons. The effective U value of the V 3*d* orbitals was 3.25 eV, while the effective U value of the Fe 3*d* orbitals was 5.3 eV. The cut-off energy was 520 eV, which was high enough for all the materials. When all the residual forces converged to less than 2×10^{-5} eV Å^{-1}, geometric relaxation was carried out.

a

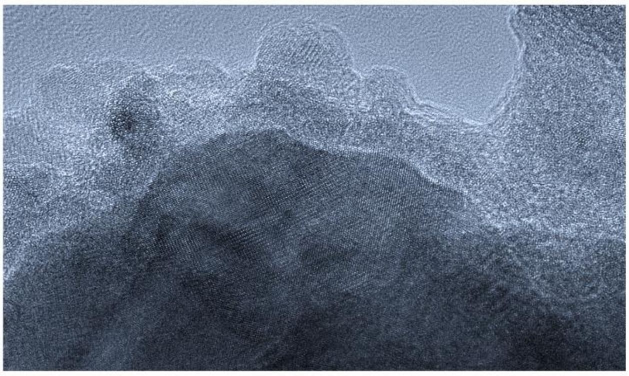

b

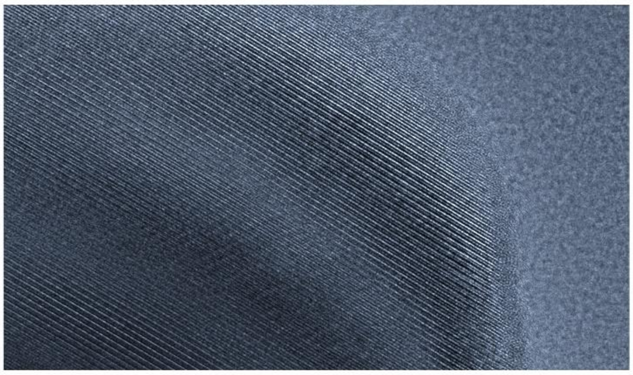

Figure S1. TEM images of the samples. (a) FeV@FeVO. (b) FeVO.

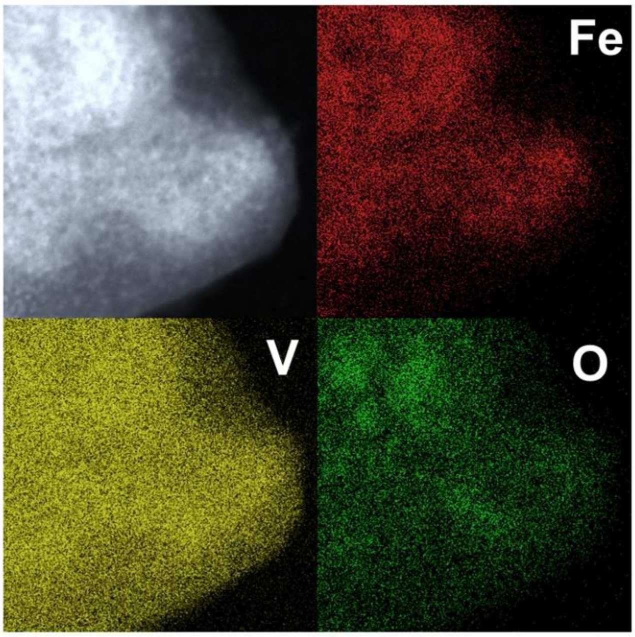

Figure S2. Energy dispersive X-ray (EDX) mapping images of the FeV@FeVO sample.

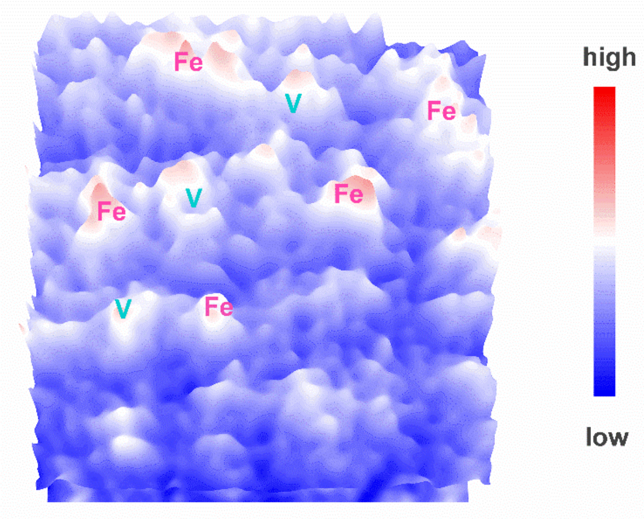

Figure S3. 3D intensity surface plot shown in dashed region 2 of the image in Fig. 1d.

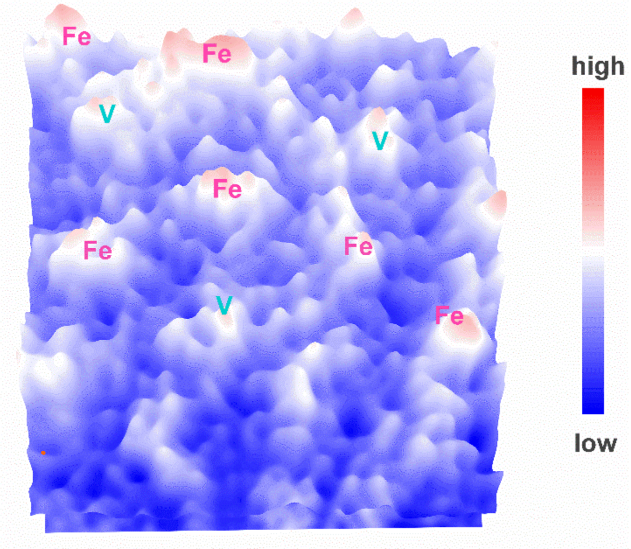

Figure S4. 3D intensity surface plot shown in dashed region 3 of the image in Fig. 1d.

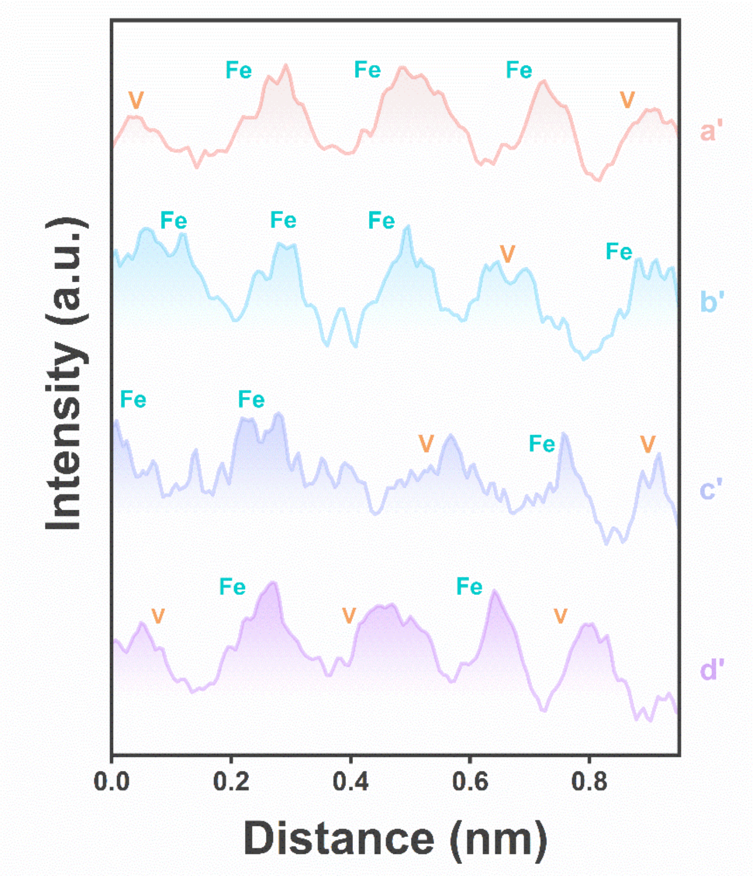

Figure S5. HAADF-STEM intensity profiles along the corresponding dashed lines in Fig. 1d.

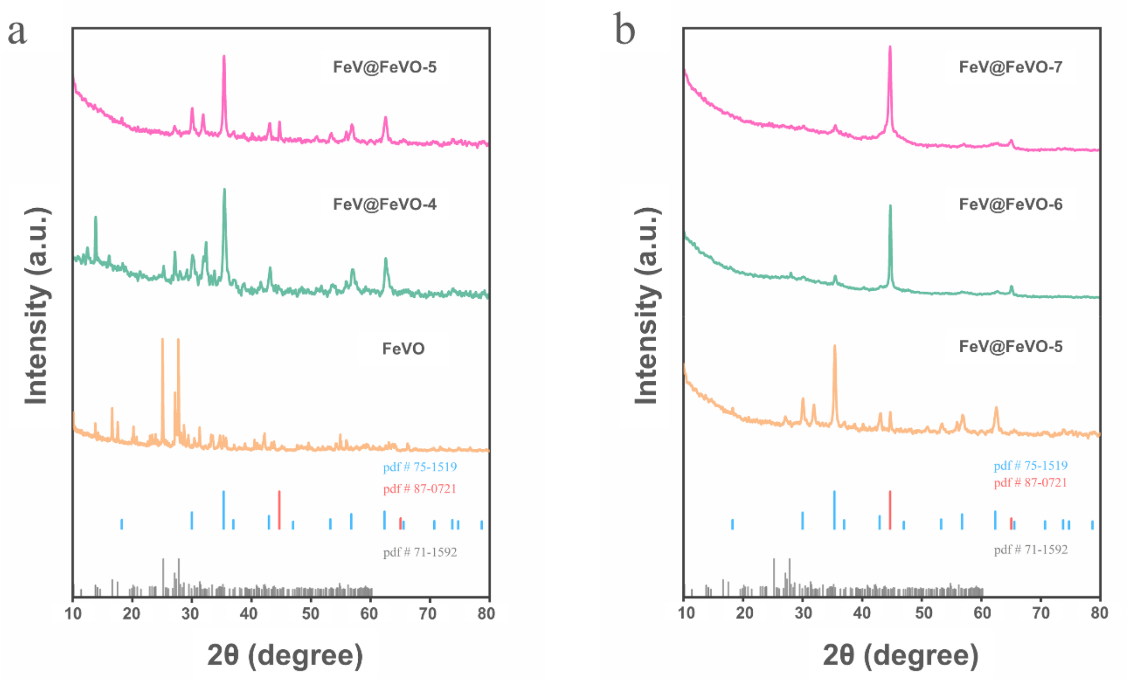

Figure S6. (a, b) XRD patterns of all the samples (pdf #71-1592 represents $FeVO_4$, pdf #75-1519 represents Fe_2VO_4, pdf #87-0721 represents Fe).

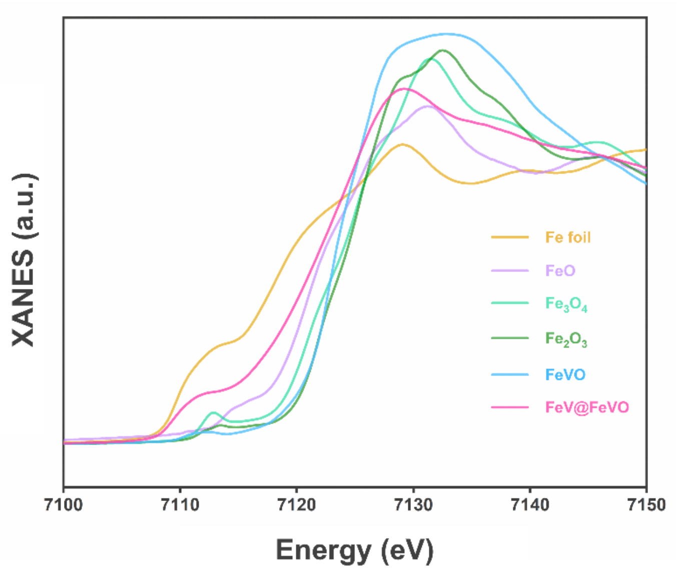

Figure S7. XANES spectra at the Fe K-edge of the FeV@FeVO and FeVO samples.

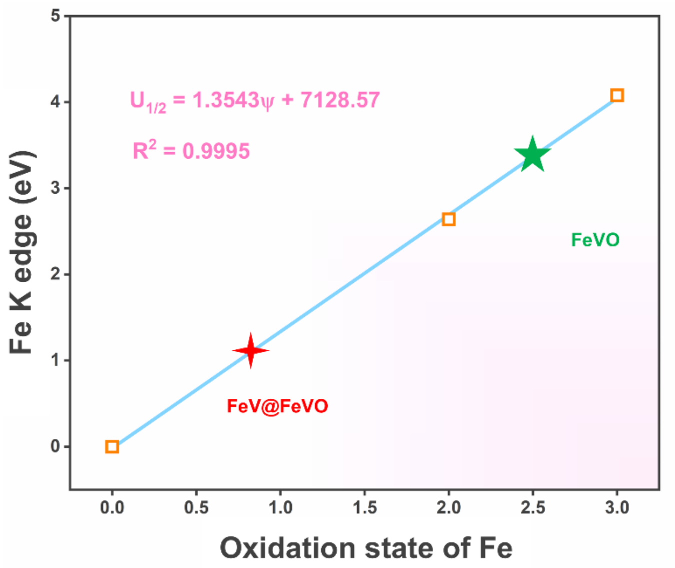

Figure S8. The mean chemical valence of Fe species in the FeV@FeVO and FeVO samples.

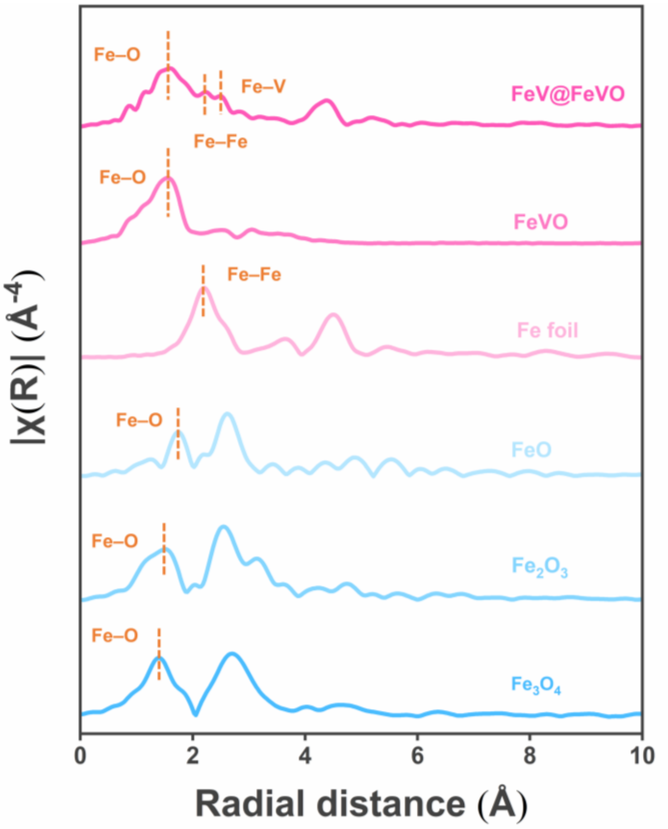

Figure S9. FT-EXAFS spectra at the Fe K-edge of the FeV@FeVO and FeVO samples.

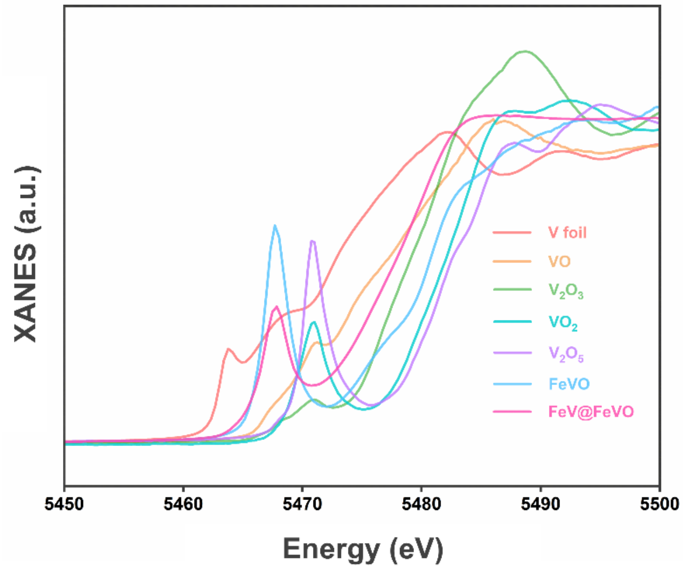

Figure S10. XANES spectra at the Fe K-edge of the FeV@FeVO and FeVO samples.

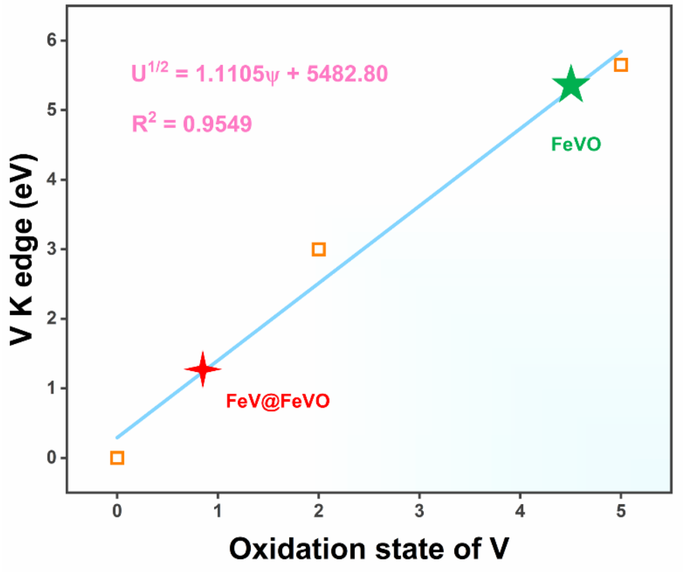

Figure S11. The mean chemical valence of V species in the FeV@FeVO and FeVO samples.

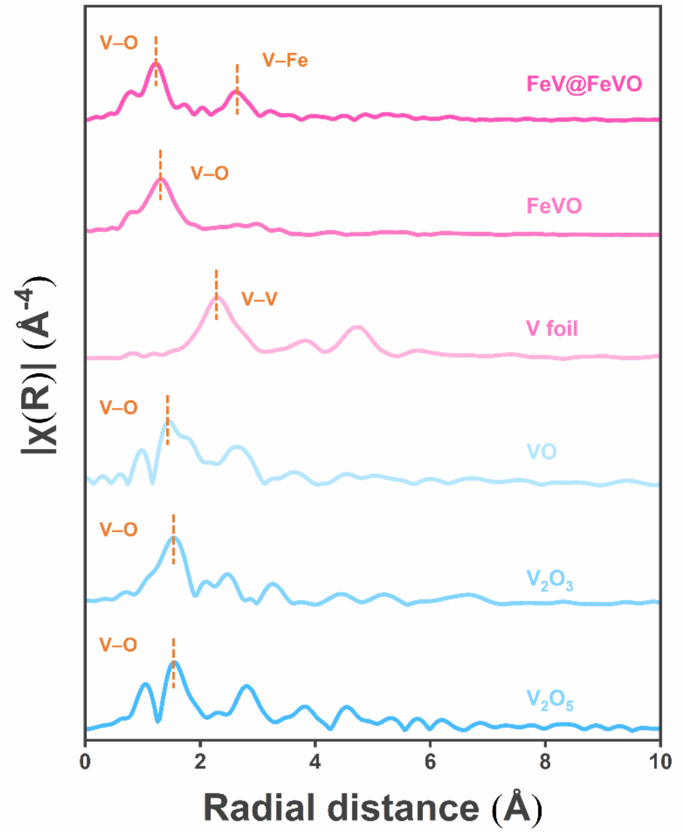

Figure S12. FT-EXAFS spectra at the V K-edge of the FeV@FeVO and FeVO samples.

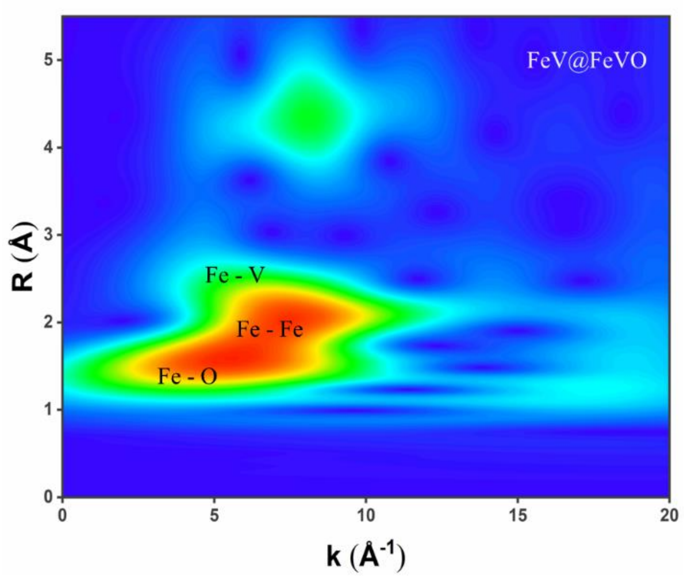

Figure S13. Wavelet transformation at the Fe K-edge of FeV@FeVO based on the k^2-weighted EXAFS signal.

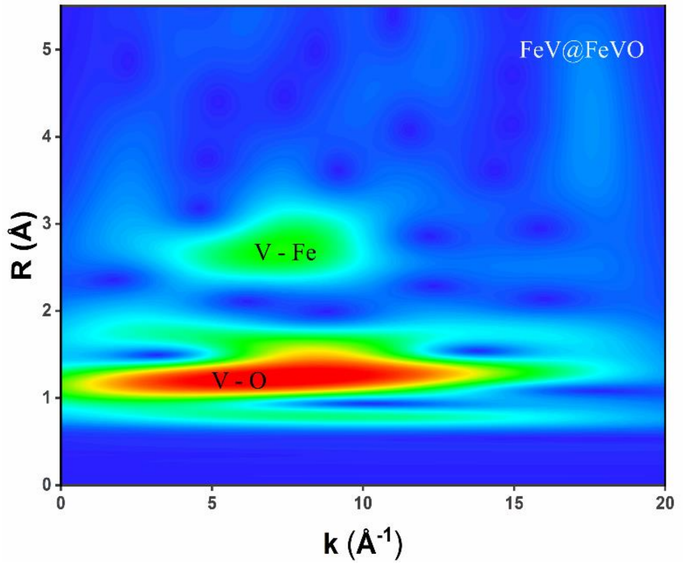

Figure S14. Wavelet transformation at the V K-edge of FeV@FeVO based on the k^2-weighted EXAFS signal.

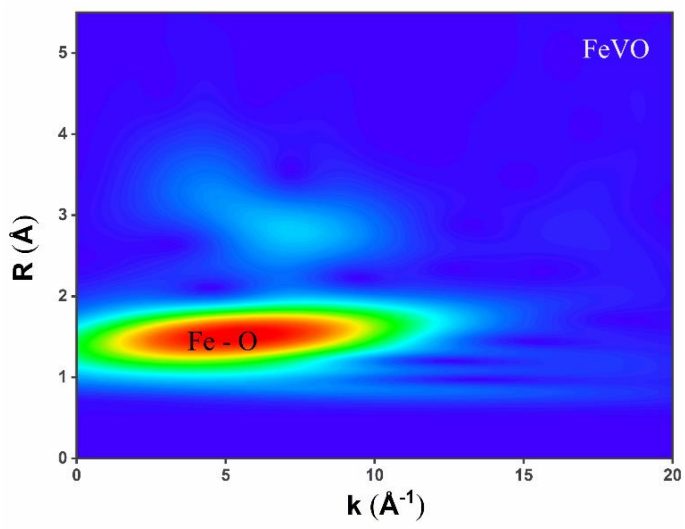

Figure S15. Wavelet transformation at the Fe K-edge of FeVO based on the k^2-weighted EXAFS signal.

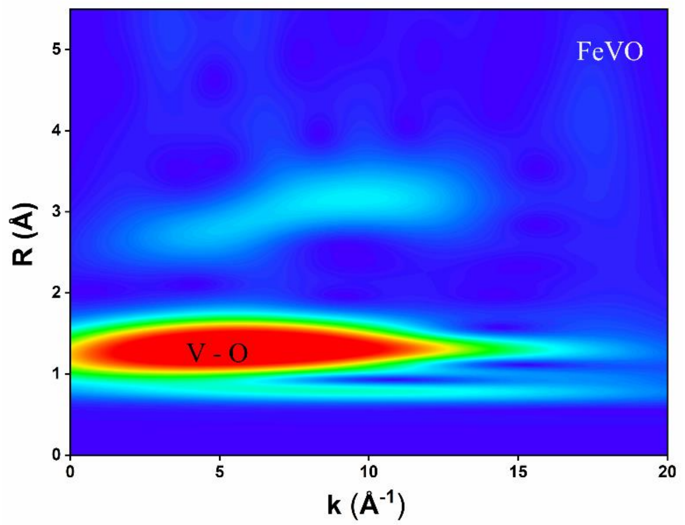

Figure S16. Wavelet transformation at the V K-edge of FeVO based on the k^2-weighted EXAFS signal.

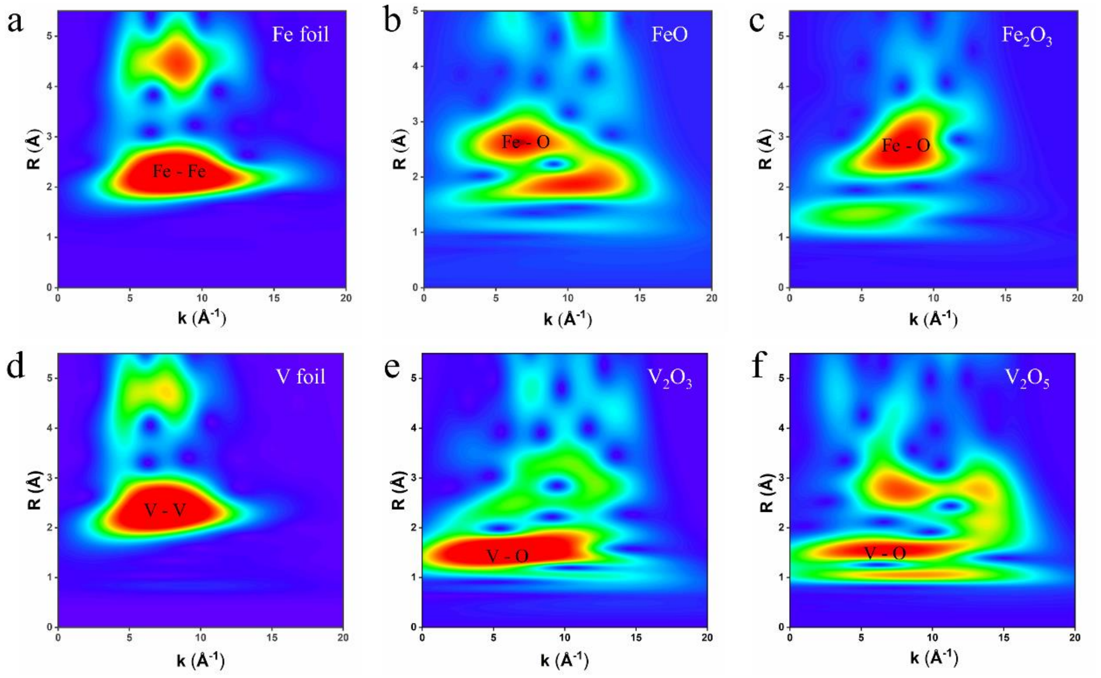

Figure S17. Wavelet transformation of the samples based on the k^2-weighted EXAFS signal. (a) Fe foil. (b) FeO. (c) Fe_2O_3. (d) V foil. (e) V_2O_3. (f) V_2O_5.

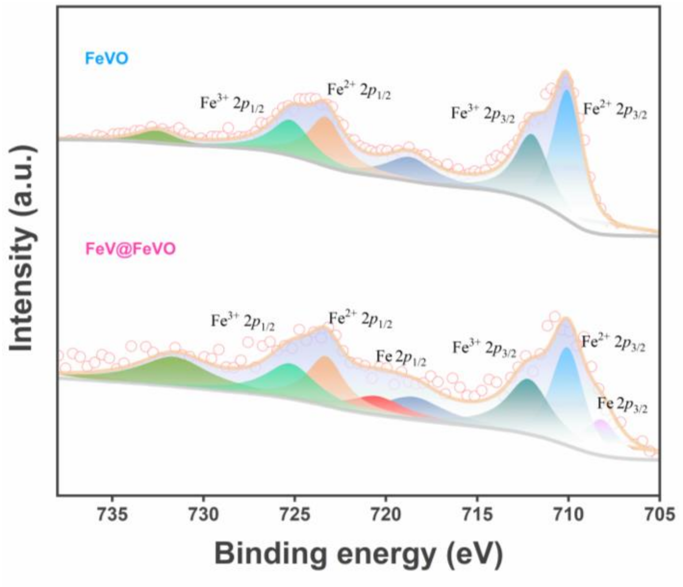

Figure S18. Fe 2*p* XPS spectra of FeVO and FeV@FeVO.

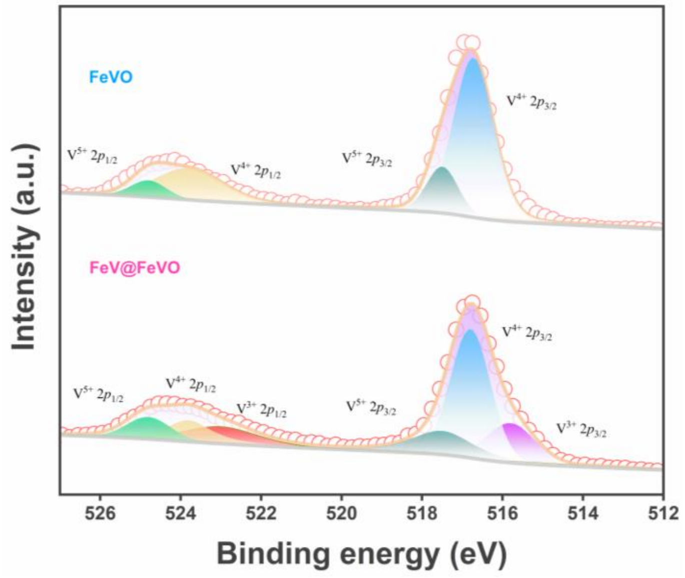

Figure S19. V 2*p* XPS spectra of FeVO and FeV@FeVO.

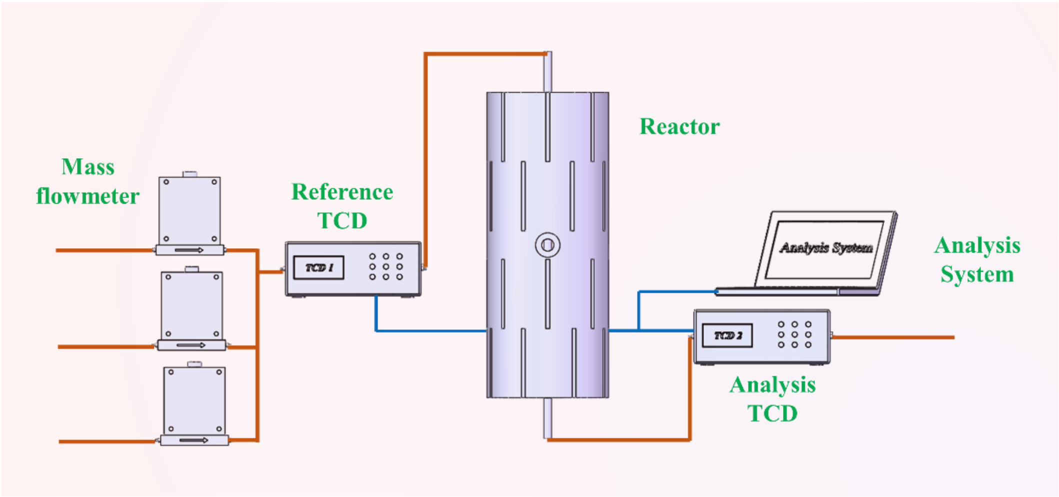

Figure S20. Schematic diagram of the photoexcited desorption mechanism.

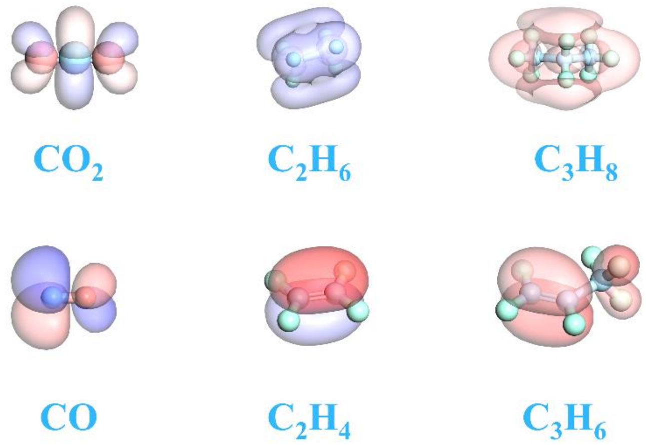

Figure S21. HOMO for CO_2, CO, C_2H_6, C_2H_4, C_3H_8 and C_3H_6.

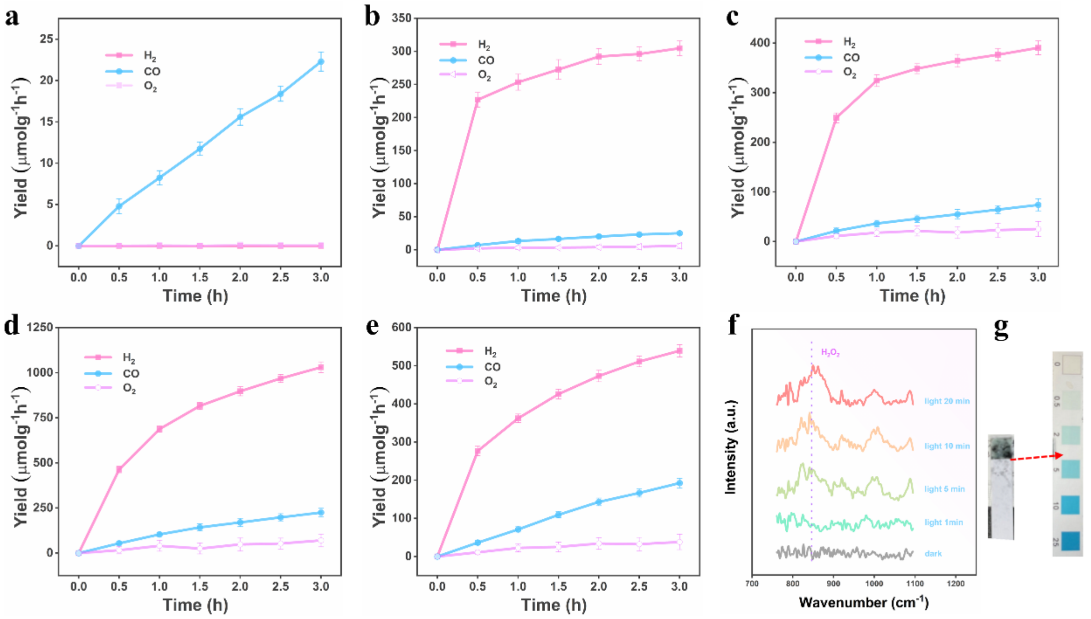

Figure S22. Photocatalytic performances and DRIFT spectra of the photocatalysts. (a) FeVO. (b) FeV@FeVO-4. (c) FeV@FeVO-5. (d) FeV@FeVO. (e) FeV@FeVO-7. (f) *In situ* DRIFT spectra over FeV@FeVO under illumination with a 300 W Xe lamp. (g) Colorimetric analysis of H_2O_2 generated via photocatalytic CO_2 reduction over FeV@FeVO.

The following steps can be used to provide a detailed explanation of the reaction process of photocatalytic carbon dioxide reduction to syngas.

Reduction steps:

(1) $CO_2 + 2e^- + H_2O \rightarrow CO + 2OH^-$

(2) $2H_2O + 2e^- \rightarrow H_2 + 2OH^-$

Oxidation steps:

(3) $2OH^- + 2h^+ \rightarrow H_2O_2$

(4) $4OH^- + 4h^+ \rightarrow O_2 + 2H_2O$

Total steps:

(5) $CO_2 + 3H_2O \rightarrow CO + H_2 + 2H_2O_2$

(6) $CO_2 + H_2O \rightarrow CO + H_2 + O_2$

According to the above reaction steps, we can obtain a deeper understanding of the entire reaction system. The reduction products are mainly syngas (CO and H_2), and the ratio of syngas can be altered by changing the synthesis conditions of the single-atom alloy catalyst. On the other hand, the oxidation products are mainly O_2 and H_2O_2. revealed that a small amount of O_2 can be detected (Figs S22a–e). In addition, the signal at approximately 870 cm^{-1} can corresponds to the H_2O_2 absorbed on the surface of FeV@FeVO (Fig. S22f), which shows that a certain amount of H_2O_2 can be generated during the reaction and adsorbed on the surface of the catalyst. The colorimetric transition of the H_2O_2 detection test paper (Fig. S22g) further verified H_2O_2 as an oxidation product.

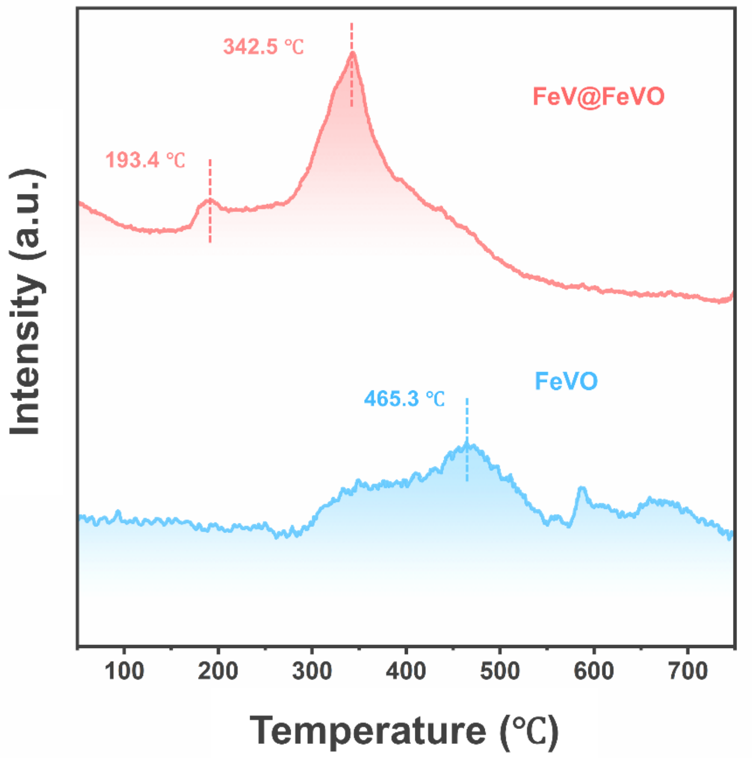

Figure S23. CO-TPD test of the samples.

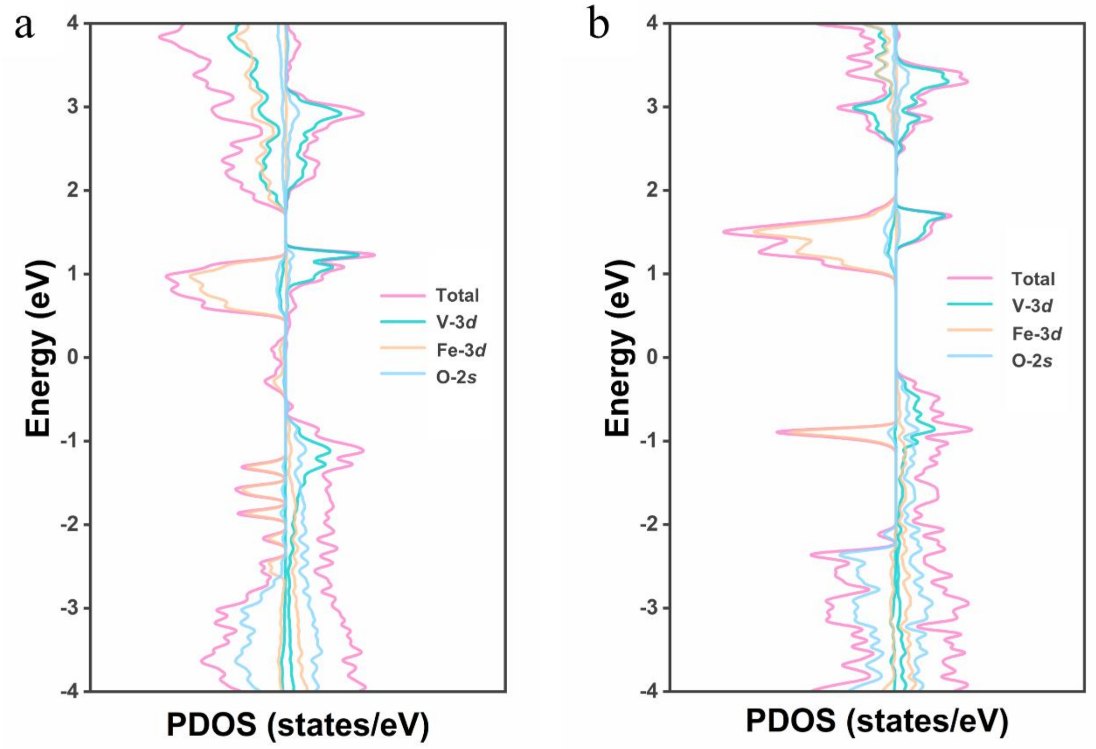

Figure S24. Partial density of states (PDOS) of (a) FeV@FeVO and (b) FeVO.

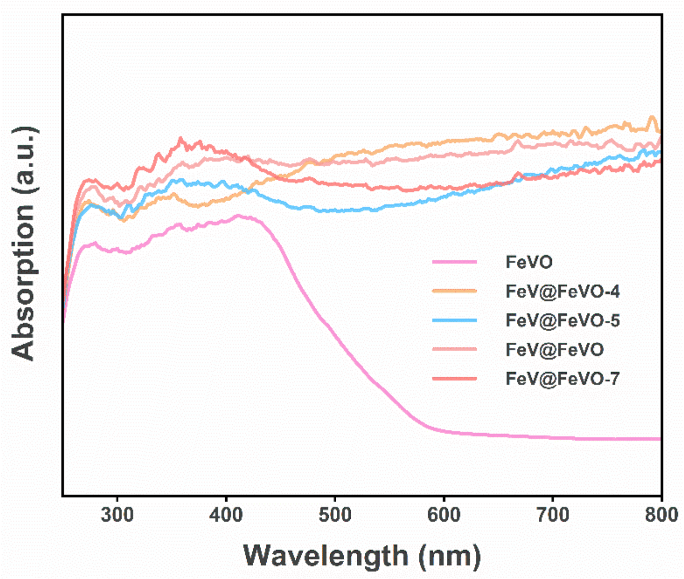

Figure S25. UV−vis absorption spectra of the synthesized samples.

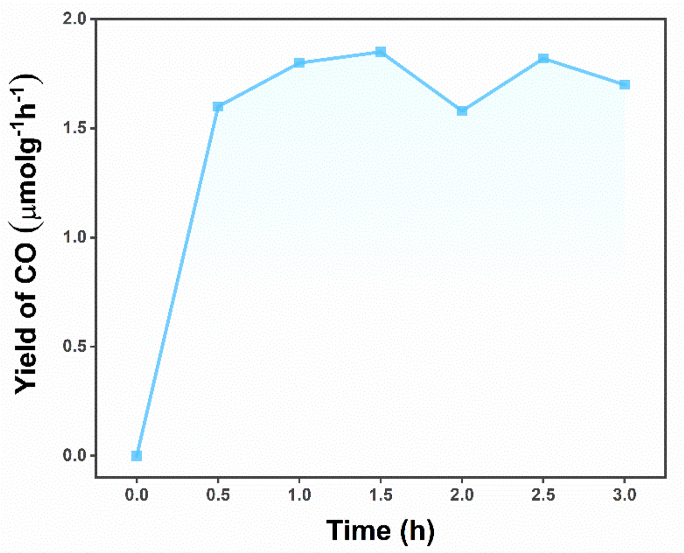

Figure S26. Photocatalytic CO production as a function of light irradiation time for FeV@FeVO during the reaction of CO_2 and H_2.

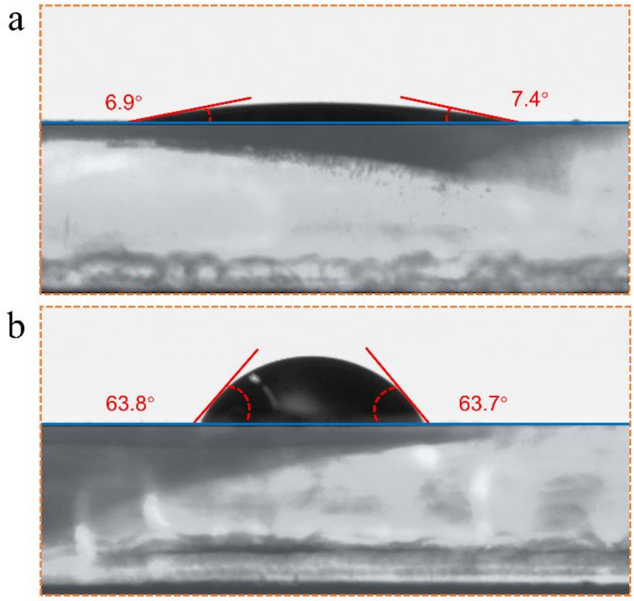

Figure S27. Water-droplet contact angle tests of (a) FeV@FeVO, and (b) FeVO.

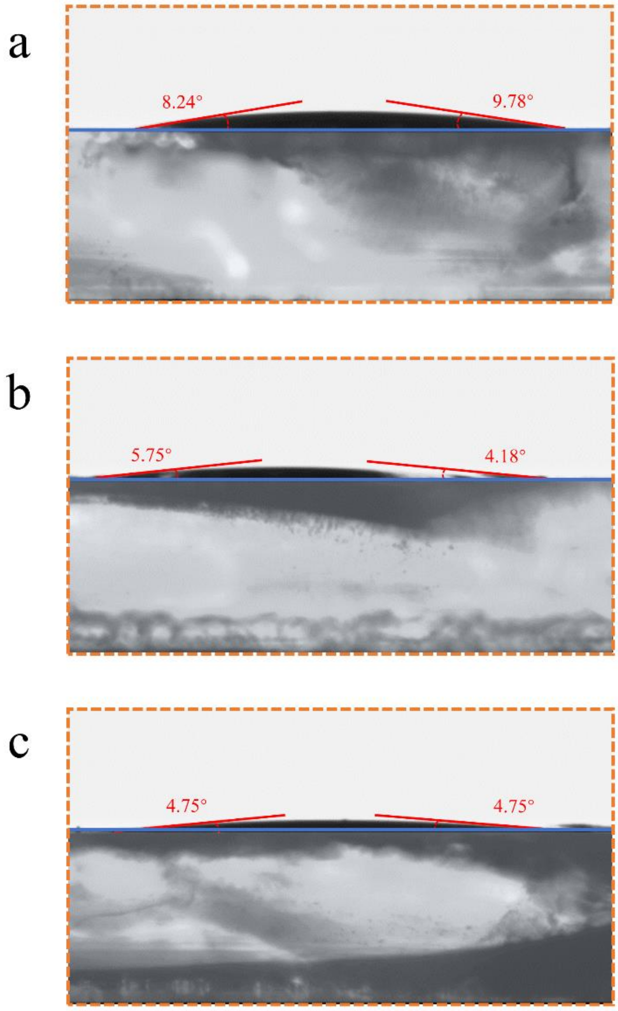

Figure S28. Water-droplet contact angle tests of (a) FeV@FeVO-4, (b) FeV@FeVO-5 and (c) FeV@FeVO-7.

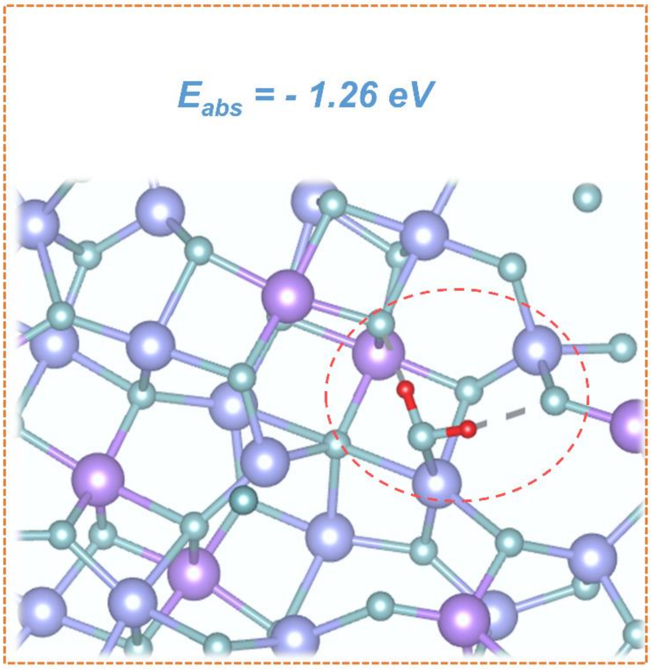

Figure S29. Structure of H_2O on the FeV@FeVO sample.

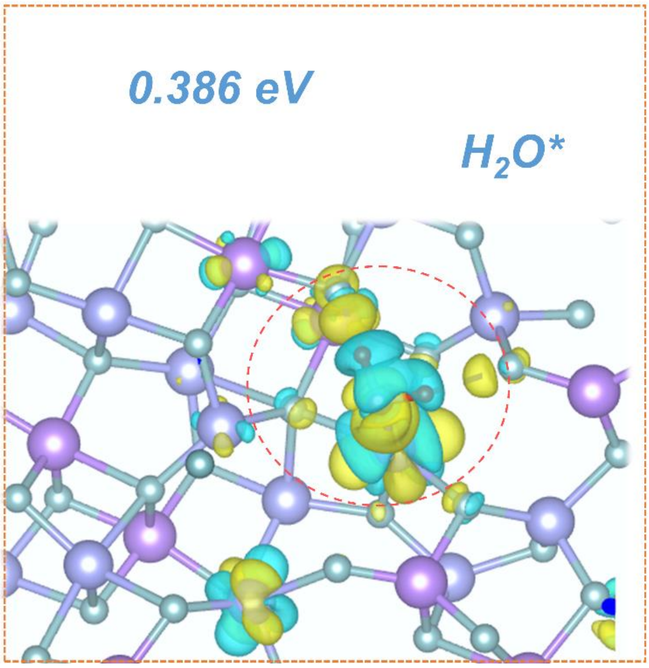

Figure S30. Charge density of H_2O on the FeV@FeVO sample.

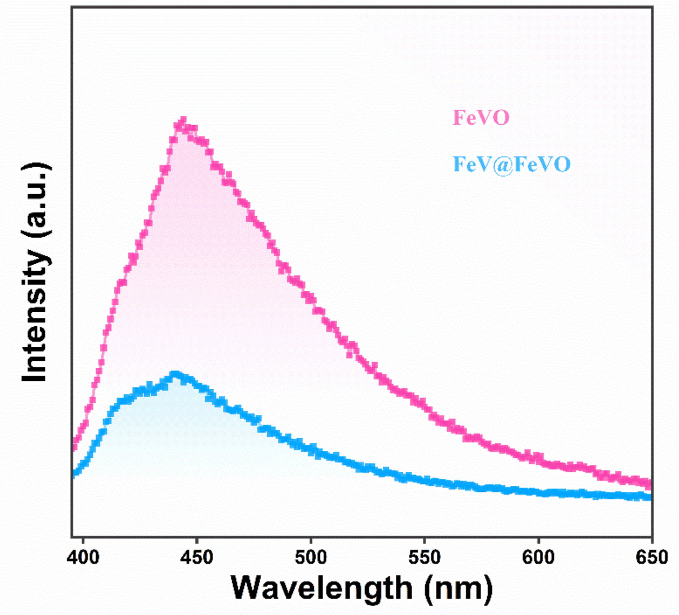

Figure S31. Steady-state PL spectra of FeVO and FeV@FeVO.

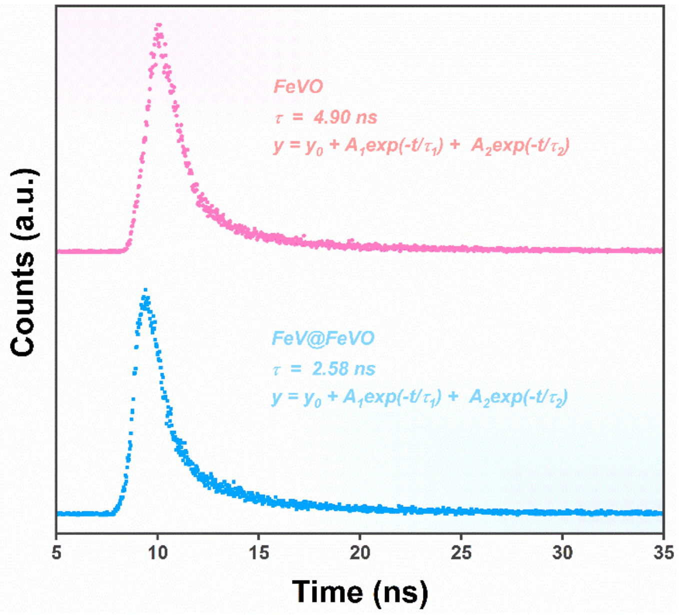

Figure S32. Time-resolved fluorescence decay spectroscopy of FeVO and FeV@FeVO.

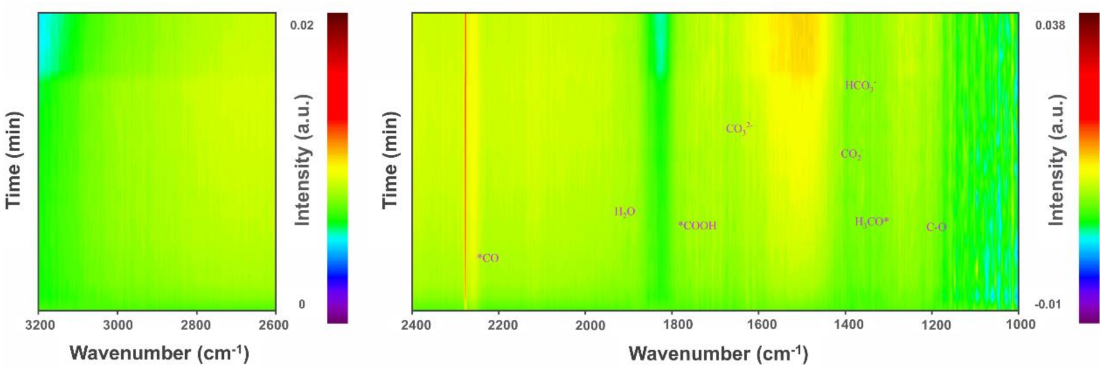

Figure S33. *In situ* DRIFTS of FeV@FeVO-4 under illumination with a 300 W Xe lamp (the test duration is 30 minutes).

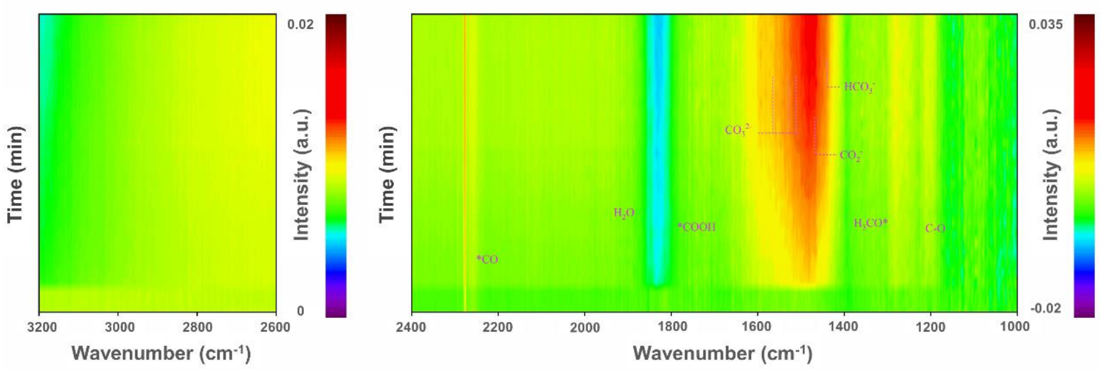

Figure S34. *In situ* DRIFTS of FeV@FeVO-5 under illumination with a 300 W Xe lamp (the test duration is 30 minutes).

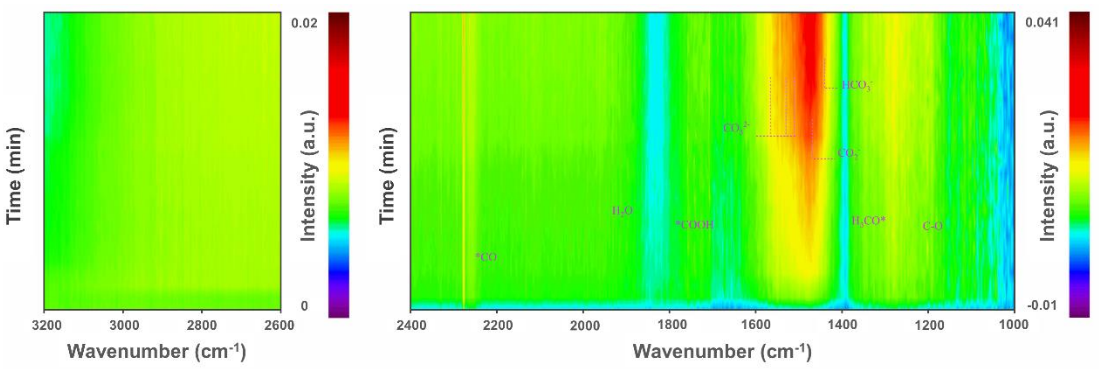

Figure S35. *In situ* DRIFTS of FeV@FeVO-7 under illumination with a 300 W Xe lamp (the test duration is 30 minutes).

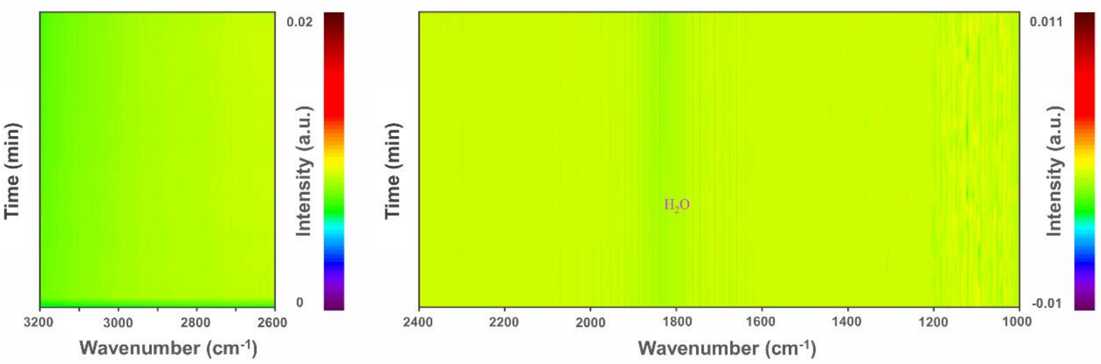

Figure S36. *In situ* DRIFTS of FeVO under illumination with a 300 W Xe lamp (the test duration is 30 minutes).

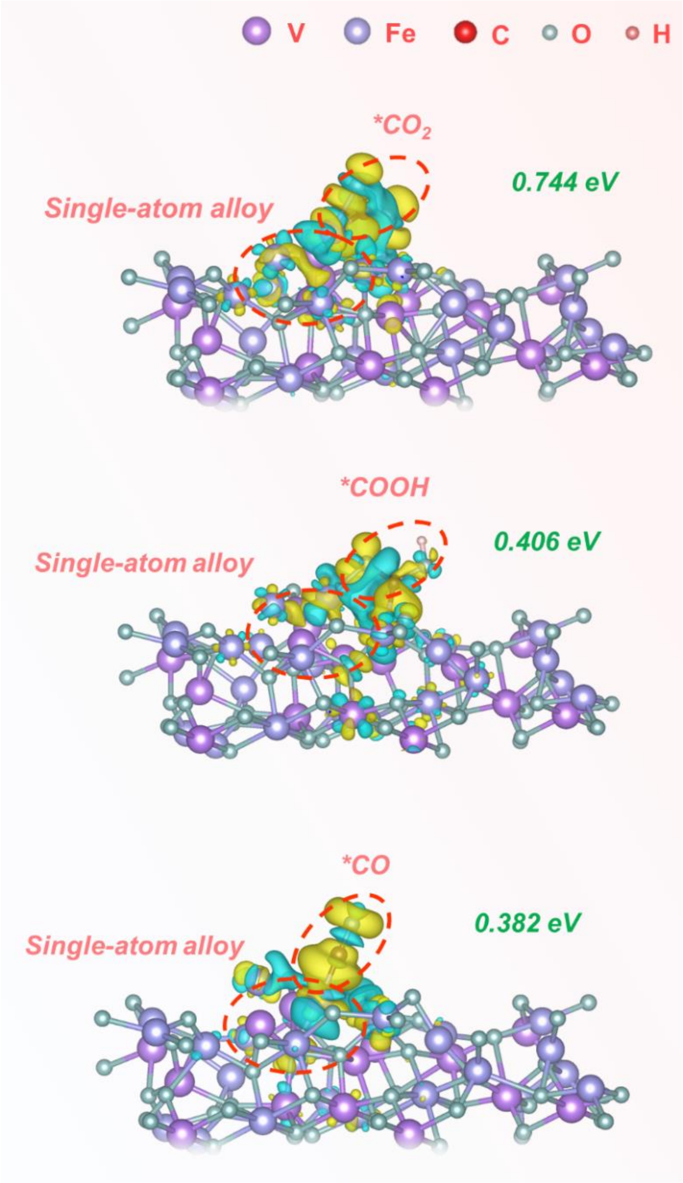

Figure S37. Charge density of $^*CO_2$, *COOH and *CO in the FeV@FeVO model.

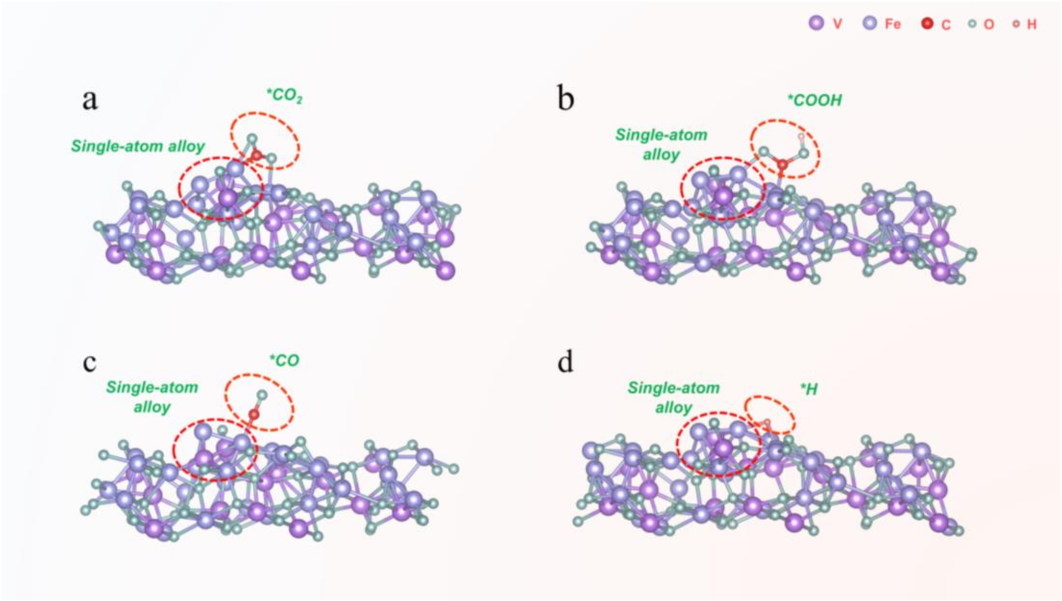

Figure S38. Adsorption models of molecules on the FeV@FeVO sample. (a) $*CO_2$. (b) *COOH. (c) *CO. (d) *H.

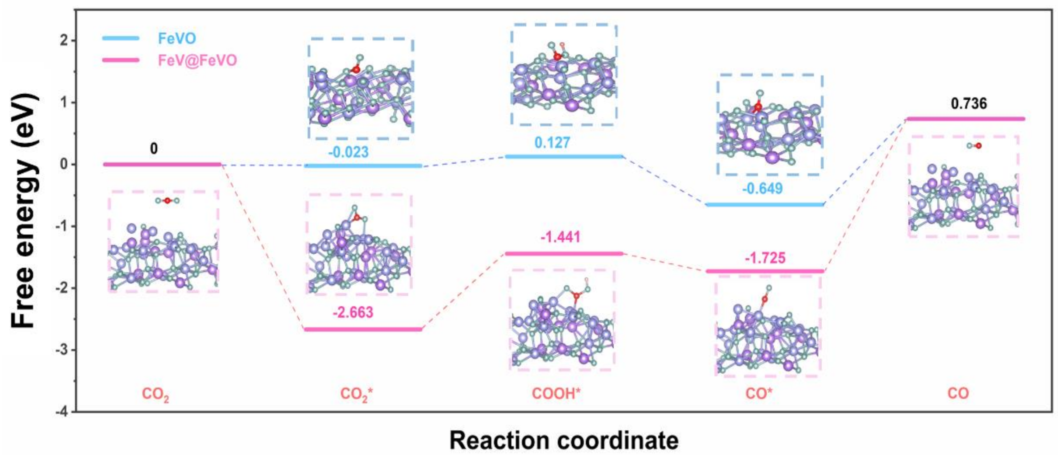

Figure S39. Free-energy diagrams of the C1 pathway on FeVO and FeV@FeVO.

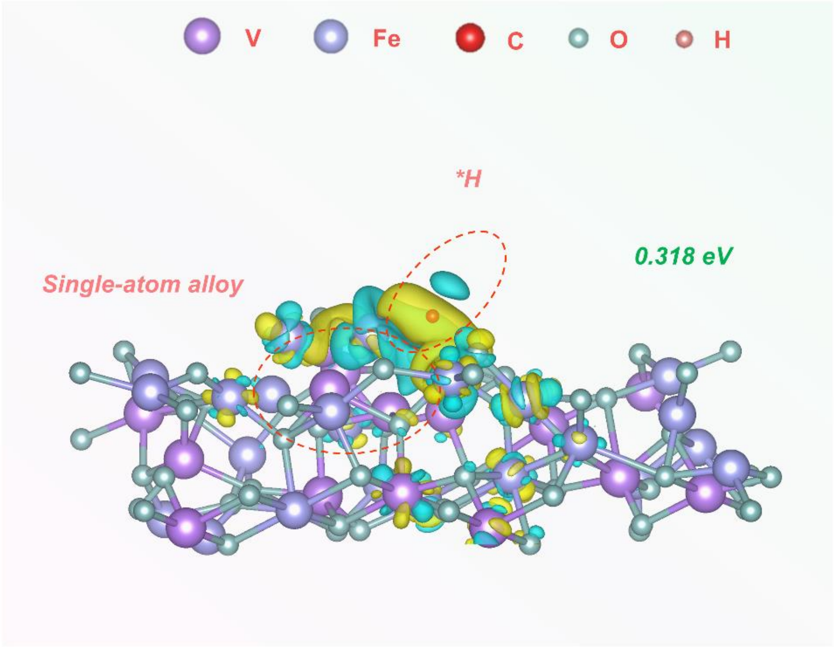

Figure S40. Charge density of *H in the FeV@FeVO model.

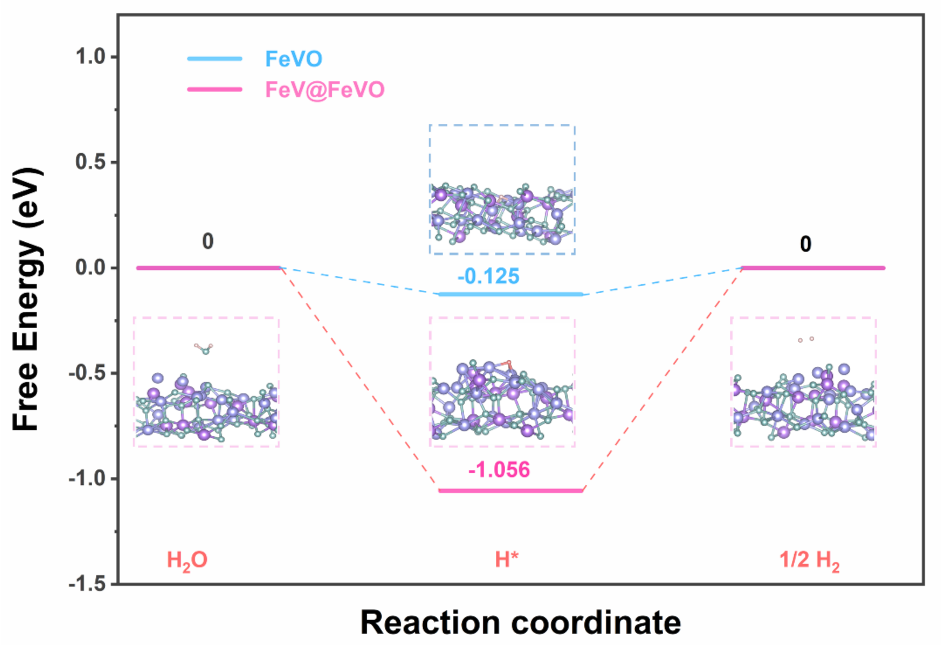

Figure S41. Free-energy diagrams of the H pathway on FeVO and FeV@FeVO.

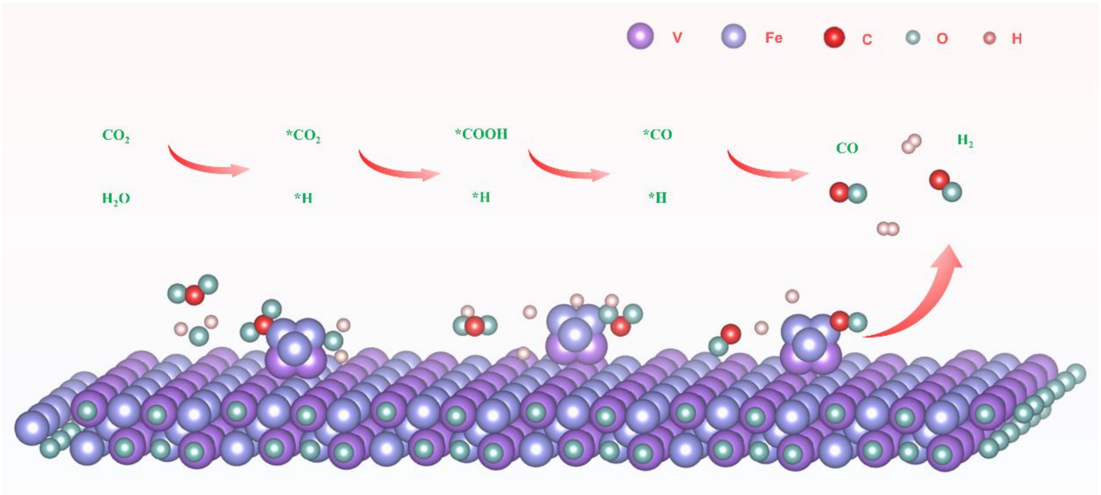

Figure S42. Schematic diagram of the mechanism of photocatalytic CO_2 reduction over FeV@FeVO.

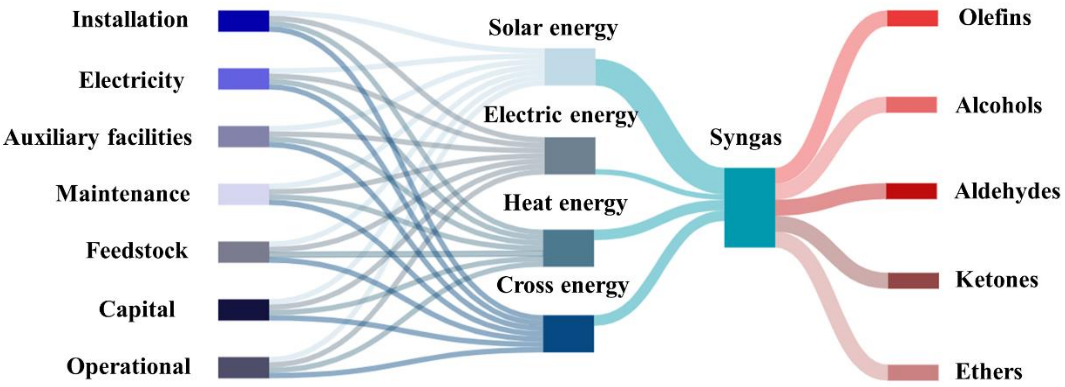

Figure S43. Sankey diagram of the upstream cost and downstream target products of syngas.

Table S1. Statistics of the LUMO and HOMO.

**Molecule**	**LUMO/eV**	**HOMO/eV**
CO_2	−0.39	−8.84
CO	−1.66	−8.67
C_2H_6	2.28	−7.88
C_2H_4	−0.24	−6.17
C_3H_8	−1.91	−7.56
C_3H_6	0	−5.71

Table S2. Comparison with representative systems for syngas production.

No.	Catalyst	Process	Performance	Noble metal	Sacrifice agents	Ref
1	Ni/Fe–N–C	EC	j > 39.33 mA cm^{-2} (−0.18 V_{RHE})	no	no	[1]
2	Ni-hG	EC	j = −14.7 mA cm^{-2} (−1.0 V_{RHE})	no	no	[2]
3	ZnO	EC	j = 40 mA cm^{-2}	no	no	[3]
4	CoPc/NiPc	EC	j > 74 mA cm^{-2} (−0.1 V_{RHE})	no	no	[4]
5	F-γ-In_2Se_3/CP	EC	j = 90.1 mA cm^{-2} (−2.3 V_{RHE})	no	no	[5]
6	PdAg	EC	j = 1.85 mA cm^{-2} (−0.9 V_{RHE})	Pd,Ag	no	[6]
7	AgP_2	EC	j = −3.2 mA cm^{-2} (−0.11 V_{RHE})	Ag	no	[7]
8	ZnLa-1/CN	EC	j = −2.5 mA cm^{-2} (−1.0 V_{RHE})	no	no	[8]
9	Fe-Ni-DASs	EC	j = −150 mA cm^{-2} (−1.2 V_{RHE})	no	no	[9]
10	Cu_2O-SnOx	PEC	Faradaic efficiency of 90.32% (−0.35 V_{RHE})	no	no	[10]
11	a-Si/TiO_2/Au	PEC	6 mA cm^{-2} at 0.2 V_{RHE}	Au	no	[11]
12	Pt-TiO_2/GaN/n+-p Si	PEC	0.87% (solar-to-syngas efficiency)	Pt	no	[12]
13	Ti|IrO_x	PEC	solar-to-CO efficiency of 17%	Ir	no	[13]
14	ZnSe/NiCycP	PEC	CO = 0.14 μmol/cm^2 h, H_2 = 0.3 μmol/cm^2 h	no	no	[14]
15	BP/ZIS	PC	CO = 4.5 μmol h^{-1}, H_2 = 8.5 μmol h^{-1}	no	TEOA	[15]
16	Co9S8@Cd0.8Zn0.2S -DETA	PC	CO = 4.7 mmol/g h, H_2 = 1.2 mmol/g h	no	TEOA	[16]
17	TiO_2-Co(terpyridine)$_2$	PC	CO = 0.22 mmol/g h, H_2 = 0.15 mmol/g h	no	TEOA	[17]
18	Cu_2S@ROH-$NiCo_2O_3$	PC	CO = 7.1 mmol/g h, H_2 = 2.8 mmol/g h	Cu	TEOA	[18]
19	Ni/Co_3O_4	PC	CO = 277.7 mmol/g h	no	TEOA	[19]
20	CdS/CNT/CoPc	PC	CO = 6.3 mmol/g h	no	TEOA	[20]
21	RGO-CoOx|$BiVO_4$|$CuGaS_2$	PC	CO = 6 μmol/g h	no	no	[21]
22	CdS-Ni(terpyS)$_2$	PC	CO = 0.16 μmol/cm^2 h, H_2 = 0.009 μmol/cm^2 h	no	TEOA	[22]
23	Fe-SAs/N-C	PC	CO = 4.5 mmol/g h, H_2 = 4.95 mmol/g h	no	TEOA	[23]
24	Fe/Ni-COFs	PC	CO = 9.06 μmol/h, H_2 = 1.16 μmol/h	no	TEOA	[24]
**25**	**FeV@FeVO**	**PC**	**CO = 107 μmol/g h, H_2 = 684.8 μmol/g h**	**no**	**no**	**This work**

'EC' indicates "electrocatalysis", 'PEC' indicates "photoelectrocatalysis", 'PC' indicates "photocatalysis", and 'TEOA' indicates "triethanolamine".

The two most popular syngas production techniques currently in use are photoelectrocatalytic and electrocatalytic CO_2 reduction, in which the syngas ratio can be adjusted by varying the input voltage. Triethanolamine is used as a hole-scavenging agent in the photocatalytic systems for syngas production from CO_2 and H_2O under illumination. However, without any scavenging agents, the photocatalytic syngas production is difficult. Our study demonstrated efficient photocatalytic syngas conversion from CO_2 and H_2O without using a sacrificial agent.

References

1. Hua YN, Zhang BW, Hao WB *et al.* Boosting CO desorption on dual active site electrocatalysts for CO_2 reduction to produce tunable syngas. *Cell Rep. Phys. Sci.* 2022; **3**: 100703.
2. Leverett J, Daiyan R, Gong LL *et al.* Designing undercoordinated Ni-N_x and Fe-N_x on holey graphene for electrochemical CO_2 conversion to syngas. *ACS Nano* 2021; **15**: 12006–12018.
3. Daiyan R, Lovell EC, Huang BS *et al.* Uncovering atomic-scale stability and reactivity in engineered zinc oxide electrocatalysts for controllable syngas production. *Adv. Energy Mater.* 2020; **10**: 2001381.
4. He Q, Liu DB, Lee JH *et al.* Electrochemical conversion of CO_2 to syngas with controllable CO/H_2 ratios over Co and Ni single-atom catalysts. *Angew. Chem. Int. Ed.* 2020; **59**: 3033–3037.
5. Yang DX, Zhu QG, Sun XF *et al.* Electrosynthesis of a defective indium selenide with 3D structure on a substrate for tunable CO_2 electroreduction to syngas. *Angew. Chem. Int. Ed.* 2020; **59**: 2354–2359.
6. Lee JH, Kattel S, Jiang Z *et al.* Tuning the activity and selectivity of electroreduction of CO_2 to synthesis gas using bimetallic catalysts. *Nat. Commun.* 2019; **10**: 3724.
7. Li H, Wen P, Itanze DS *et al.* Colloidal silver diphosphide (AgP_2) nanocrystals as low overpotential catalysts for CO_2 reduction to tunable syngas. *Nat. Commun.* 2019; **10**: 5724.

8. Liang Z, Song LP, Sun MZ *et al.* Tunable CO/H_2 ratios of electrochemical reduction of CO_2 through the Zn-Ln dual atomic catalysts. *Sci. Adv.* 2021; **7**: eabl4915.

9. Wang LX, Gao XP, Wang SC *et al.*, Axial dual atomic sites confined by layer stacking for electroreduction of CO_2 to tunable syngas. *J. Am. Chem. Soc.* 2023; **145**: 13462–13468.

10. Zhang Y, Pan DL, Tao Y *et al.* Photoelectrocatalytic reduction of CO_2 to syngas via SnO_x-enhanced Cu_2O nanowires photocathodes. *Adv. Funct. Mater.* 2021; **32**: 2109600.

11. Li CC, Wang T, Liu B *et al.* Photoelectrochemical CO_2 reduction to adjustable syngas on grain-boundary-mediated a-Si/TiO_2/Au photocathodes with low onset potentials. *Energy Environ. Sci.* 2019; **12**: 923-928.

12. Chu S, Ou PF, Ghamari P *et al.* Photoelectrochemical CO_2 reduction into syngas with the metal/oxide interface. *J. Am. Chem. Soc.* 2018; **140**: 7869–7877.

13. Boutin E, Patel M, Kecsenovity E *et al.* Photo-electrochemical conversion of CO_2 under concentrated sunlight enables combination of high reaction rate and efficiency. *Adv. Energy Mater.* 2022; **12**: 2200585.

14. Kuehnel MF, Sahm CD, Neri G *et al.* ZnSe quantum dots modified with a Ni(cyclam) catalyst for efficient visible-light driven CO_2 reduction in water. *Chem. Sci.* 2018; **9**: 2501–2509.

15. Han C, Li YH, Li JY *et al.* Cooperative syngas production and C-N bond formation in one photoredox cycle. *Angew. Chem. Int. Ed.* 2021; **60**: 7962–7970 (2021).

16. Su B, Zheng M, Lin W *et al.* S-scheme Co_9S_8@$Cd_{0.8}Zn_{0.2}S$-DETA hierarchical nanocages bearing organic CO_2 activators for photocatalytic syngas production. *Adv. Energy Mater.* 2023; **13**: 2203290.

17. Lam E, Reisner E. A TiO_2-Co(terpyridine)$_2$ photocatalyst for the selective oxidation of cellulose to formate coupled to the reduction of CO_2 to syngas.

Angew. Chem. Int. Ed. 2021; **60**: 23306–23312.

18. Li L, Dai XY, Chen DL *et al.* Steering catalytic activity and selectivity of CO_2 photoreduction to syngas with hydroxy-rich Cu_2S@R(OH)-$NiCo_2O_3$ double-shelled nanoboxes. *Angew. Chem. Int. Ed.* 2022; **61**: e202205839.
19. Qian G, Lyu WY, Zhao X *et al.* Efficient photoreduction of diluted CO_2 to tunable syngas by Ni-Co dual sites through d-band center manipulation. *Angew. Chem. Int. Ed.* 2022; **61**: e202210576.
20. Choi C, Zhao FY, Hart JL *et al.* Synergizing electron and heat flows in photocatalyst for direct conversion of captured CO_2. *Angew. Chem. Int. Ed.* 2023; **62**: e202302152.
21. Iwase A, Yoshino S, Takayama T *et al.* Water splitting and CO_2 reduction under visible light irradiation using Z-scheme systems sonsisting of metal sulfides, CoO_x-loaded $BiVO_4$, and a reduced graphene oxide electron mediator. *J. Am. Chem. Soc.* 2016; **138**: 10260–10264.
22. Kuehnel MF, Orchard KL, Dalle KE *et al.* Selective photocatalytic CO_2 reduction in water through anchoring of a molecular Ni catalyst on CdS nanocrystals. *J. Am. Chem. Soc.* 2017; **139**: 7217–7223.
23. Wang ZY, Yang J, Cao JB *et al.* Room-temperature synthesis of single iron site by electrofiltration for photoreduction of CO_2 into tunable syngas. *ACS Nano* 2020; **14**: 6164–6172.
24. Han B, Ou XW, Zhong ZQ *et al.* Rational design of FeNi bimetal modified covalent organic frameworks for photoconversion of anthropogenic CO_2 into widely tunable syngas. *Small* 2020; **16**: e2002985.
